# Immunohistochemical correlates of *TP53* somatic mutations in cancer

**DOI:** 10.18632/oncotarget.11912

**Published:** 2016-09-08

**Authors:** Balázs Murnyák, Tibor Hortobágyi

**Affiliations:** ^1^ Division of Neuropathology, Institute of Pathology, Faculty of Medicine, University of Debrecen, Debrecen, Hungary

**Keywords:** IARC TP53 Database, immunohistochemistry, TP53 mutations, cancer, p53

## Abstract

Despite controversy on the correlation between p53 accumulation and *TP53* mutational status, ihas long been used as a surrogate method for mutation analysis. The aim of our study was to characterise the IHC expression features of *TP53* somatic mutations and define their occurrence in human cancers. A large-scale database analysis was conducted in the IARC TP53 Database (R17); 7878 mutations with IHC features were retrieved representing 60 distinct tumour sites. The majority of the alterations were immunopositive (*p* <0.001). Sex was known for 4897 mutations showing a female dominance (57.2%) and females carrying negative mutations were significantly younger. *TP53* mutations were divided into three IHC groups according to mutation frequency and IHC positivity. Each group had female dominance. Among the IHC groups, significant correlations were observed with age at diagnosis in breast, bladder, liver, haematopoietic system and head & neck cancers. An increased likelihood of false negative IHC associated with rare nonsense mutations was observed in certain tumour sites. Our study demonstrates that p53 immunopositivity largely correlates with *TP53* mutational status but expression is absent in certain mutation types.Besides, describing the complex IHC expression of *TP53* somatic mutations, our results reveal some caveats for the diagnostic practice.

## INTRODUCTION

The *TP53* gene (17p13) encodes a nuclear transcription factor expressed in response to various stress signals such as DNA damage, heat shock, hypoxia, and oncogene overexpression. Upon activation, p53 maintains the integrity and stability of the genome by triggering cell-cycle arrest, DNA repair, and apoptosis [1]. The 393-amino-acid tumour-suppressor protein consists of an N-terminal transactivation domain (residues 1-42), a proline-rich domain (residues 40-92), a sequence specific core DNA binding domain (residues 103-306), a tetramerization domain (residues 307-355) and a C-terminal regulatory domain (residues 356-393) [2].

While other tumour suppressor genes are inactivated by mutations leading to absence of protein, *TP53* alterations are usually missense producing stable full-length protein [3]. Somatic *TP53* mutations primarily occur at the 175, 245, 248, 249, 273 and 282 ‘hotspot’ codons in the conserved DNA binding domain (DBD), but they can also happen in any other sites within the gene [4]. Extensive efforts have been made to study *TP53* mutation effect on prognosis, therapeutic response and its role in cancer diagnosis. It was previously reported, that p53 overexpression is a prognostic indicator in colorectal, lung, prostate, and breast carcinomas [5-7].

Fast and reliable detection is crucial for the accurate diagnostic decisions and targeted therapies [8]. There is an agreement that overexpressed p53 protein indicates the presence of *TP53* alterations [9]. Wild-type p53 is an unstable protein with a short half-life for its detection by immunohistochemistry (IHC), but mutant p53 can accumulate within tumour cells creating a stable target for IHC [10]. Although nucleotide sequencing is the gold standard to identify *TP53* mutations, due to its beneficial features IHC has long been used as a surrogate method for mutations analysis in histopathological diagnostic practice [11].

Recent tumour profiling and data sequencing databases enable to more complex research, including molecular epidemiology, clinical surveys, and structural analyses [12, 13]. The International Agency for Research on Cancer (IARC) TP53 Mutation Database (http://p53.iarc.fr/) contains data on the prevalence and patterns of more than 28000 somatic mutations in human cancers, annotations of tumour phenotype, patient characteristics, the structural and functional impact, and immunostaining of the mutations. The database includes all published *TP53* mutations, all confirmed by sequencing, published in the peer-reviewed journals or compiled in mutation data repositories [1, 14].

The aims of our study were to characterise the IHC expression features of *TP53* somatic mutations, and define their occurrences in human cancers. For this purpose, a large-scale analysis has been conducted involving the available p53 IHC and clinicopathological data in the IARC TP53 Database. Besides, describing the IHC expression characteristics of *TP53* mutations, our results revealed some caveats of using p53 IHC as a surrogate for mutation analysis.

## RESULTS

### IHC expression characteristics of somatic mutations

Altogether, p53 IHC data was available in 7878 mutations, representing 26.5% of the 29711 *TP53* somatic mutations in the IARC Database. The IHC staining was positive in 6026 mutations (76.5%), whereas 1852 mutations (23.5%) were negative by IHC (*p* < 0.001). A key purpose of this study was to provide a complex characterisation of IHC patterns of *TP53* mutations. Regarding mutation types, single nucleotide alterations were predominantly IHC positive at a range between 74.9% and 84.6%; tandem (93.8%) and complex mutations were also positive, whereas deletions and insertions were mostly negative (57% and 59.7%, respectively). As expected, 88% of missense mutations were p53 IHC positive, whereas 71.2% of nonsense mutations were negative for IHC (*p* < 0.001).

Almost eighty percent (77.3%) of the mutations were positive within the coding regions (e.g. exons 2-11), whereas the majority of alterations in the non-coding sequences were negative (56.5%) for IHC (*p* = 0.001) (Supplemental Figure S1). Mutations outside and within the CpG island regions were positive in 83.3% and 73.3%, respectively (*p* < 0.001). *TP53* mutations were positive in non-splice site sequences as well as in alternative and cryptic sites (77%, 83.1% and 74.2%, respectively), but 64.3% of the mutations were negative in the consensus splice sites. The IHC expression patterns were diverse among the structural motifs of mutated p53.

Mutations were unequivocally positive in L1/S/H2 (86.2%), L2/L3 loops (84.3%), and NDBL/beta-sheets motifs (70.1%). Furthermore, alterations were positive in the C-terminal (55.6%), C-terminal/NLS (58.1%), and N-terminal/Transactivation/NES (60%) motifs. On the other hand, *TP53* mutations were mostly negative in the N-terminal (76.5%), N-terminal/Transactivation (60.6%), and SH3-like/Proline-rich (59.4%) motifs. The structural motifs of 224 mutations were non-available (Table 1).

**Table 1:** Immunohistochemical expression characteristics of *TP53* mutations

Mutation features	p53 immunohistochemistry					*p-value*^a^
	Total (*n* = 7878)	Negative (%) (*n* = 1852)		Positive (%) (*n* = 6026)		
**Exons/introns**						
exons	7690	1745	(22.69)	5945	(77.31)	**0.001**
introns	186	105	(56.45)	2086	(43.55)	
NA	2	2	(100.00)	0	(0.00)	
**CpG site**						
no	5373	1433	(26.67)	3940	(73.33)	**< 0.0001**
yes	2505	419	(16.73)	2086	(83.27)	**< 0.0001**
**Splice site**						
alternative	71	12	(16.90)	59	(83.10)	0.1872
consensus	112	72	(64.29)	40	(35.71)	**< 0.0001**
criptic	62	16	(25.81)	46	(74.19)	0.6684
no	7633	1752	(22.95)	5881	(77.05)	**< 0.0001**
**Type**						
Single nucleotide mutations^b^	6860	1324	(19.30)	5536	(80.70)	**<0.0001**
tandem	97	6	(6.19)	91	(93.81)	**5.16E-05**
complex	48	18	(37.50)	30	(72.50)	**0.0219**
deletion	667	380	(56.97)	287	(43.03)	**<0.0001**
insertion	196	117	(59.69)	79	(40.31)	**<0.0001**
NA	10	7	(70.00)	3	(30.00)	**0.000522**
**Effects**						
frameshift	677	451	(66.62)	226	(33.38)	**<0.0001**
intronic	53	25	(47.17)	28	(52.83)	**4.58E-05**
large deletion	2	2	(100.00)	0	(0.00)	**0.0107**
missense	5889	707	(12.01)	5182	(87.99)	**<0.0001**
nonsense	552	393	(71.20)	159	(28.80)	**<0.0001**
silent	367	136	(37.06)	231	(62.94)	**3.64E-10**
splice	131	86	(65.65)	45	(34.35)	**<0.0001**
other	177	44	(24.86)	133	(75.14)	0.6683
NA	30	8	(26.67)	22	(73.33)	0.6828
**Structural motif**						
C-terminal	234	104	(44.44)	130	(55.56)	**<0.0001**
C- terminal/NLS	31	13	(41.94)	18	(58.06)	**0.0153**
C- terminal/tetramerization	101	39	(38.61)	62	(61.39)	**3.15E-04**
L1/S/H2	1693	234	(13.82)	1459	(86.18)	**<0.0001**
L2/L3	2765	434	(15.70)	2331	(84.30)	**<0.0001**
N- terminal	34	26	(76.47)	8	(23.53)	**<0.0001**
N- terminal/Transactivation	33	20	(60.61)	13	(39.39)	**<0.0001**
N- terminal/Transactivation/ NES	5	2	(40.00)	3	(60.00)	0.3844
NDBL/β-sheets	2657	795	(29.92)	1862	(70.08)	<0.0001
SH3-like/Proline-rich	101	60	(59.41)	41	(40.59)	<0.0001
NA	224	125	(55.80)	99	(44.20)	<0.0001

a Comparisons between groups were performed with Pearson's Chi-square (χ^2^), Significant *p*-values are marked in bold;

b Containing the following point mutations: A:T>C:G, A:T>G:C, A:T>T:A, G:C>A:T, G:C>A:T at CpG, G:C>C:G and G:C>T:A)

**Figure 1:**
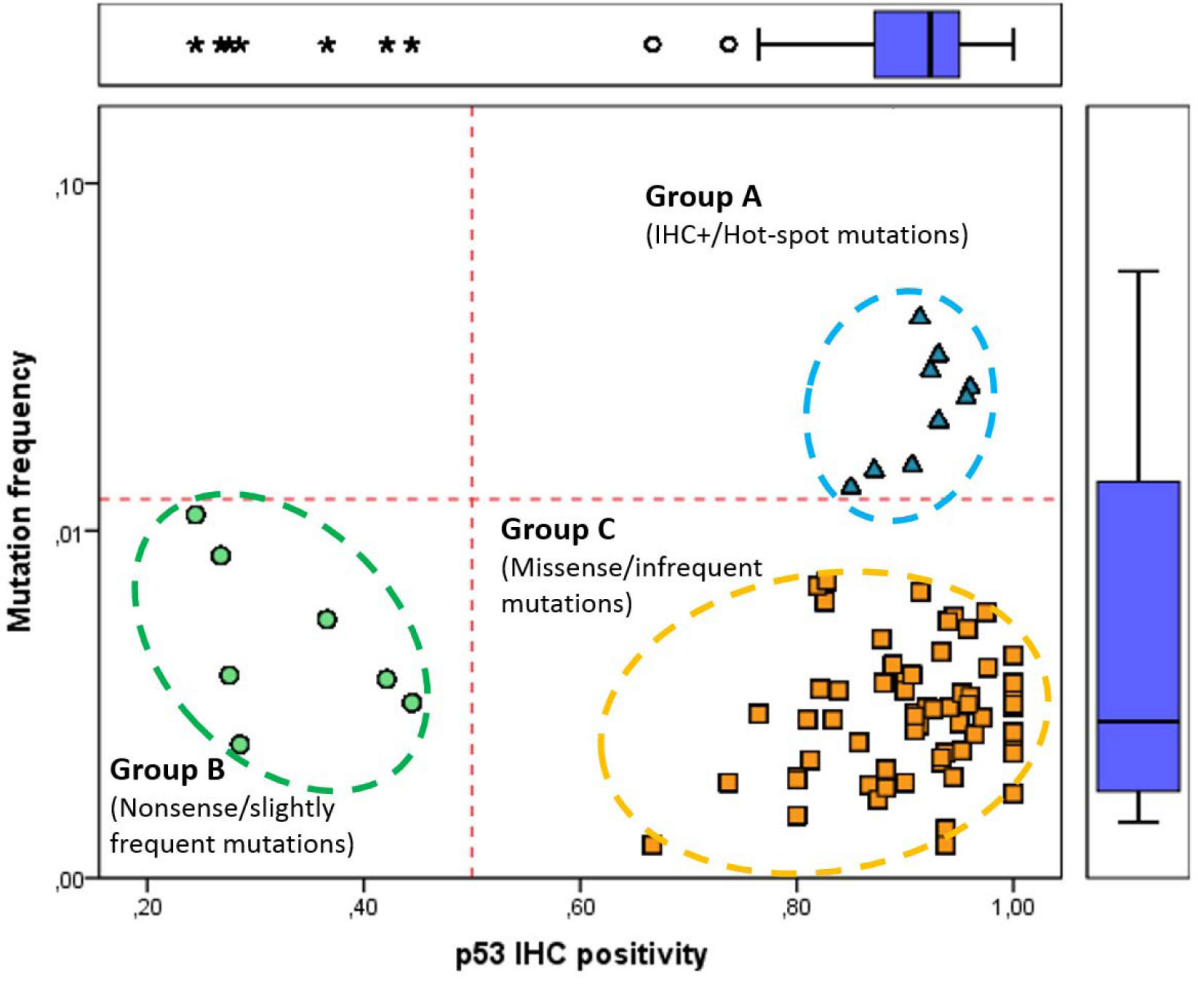
Classification of *TP53* somatic mutations based on frequency and IHC positivity in IARC TP53 Database Somatic *TP53* alterations were categorized into three groups: ‘hot spot’ mutations (marked with blue triangle) are frequent in Group A (mutation frequency ≥ 0.013 and IHC positivity ≥ 0.85); common nonsense mutations (blue circles) are in Group B (mutation frequency ≤ 0.011 and IHC positivity ≤ 0.44); less frequent and mostly positive missense mutations (yellow square) fall into Group C (mutation frequency ≤ 0.072 and IHC positivity ≥ 0.74). Each data point represents distinct *TP53* mutations and only individual mutations with 15 or more IHC results were considered.

### IHC expression patterns of individual somatic *TP53* mutations

Our other purpose was to describe the IHC expression profile of individual *TP53* mutations and compare their clinico-pathological features in various tumour sites. First, we estimated the frequencies of each 1778 individual *TP53* mutations in the IARC database. Second, the IHC results were retrieved from the database. IHC expression patterns were available of 1139 individual *TP53* mutations. Finally, we associated the alterations with their expression patterns. IHC data and frequencies of *TP53* somatic mutations are summarized in Supplemental Table S1.

**Figure 2:**
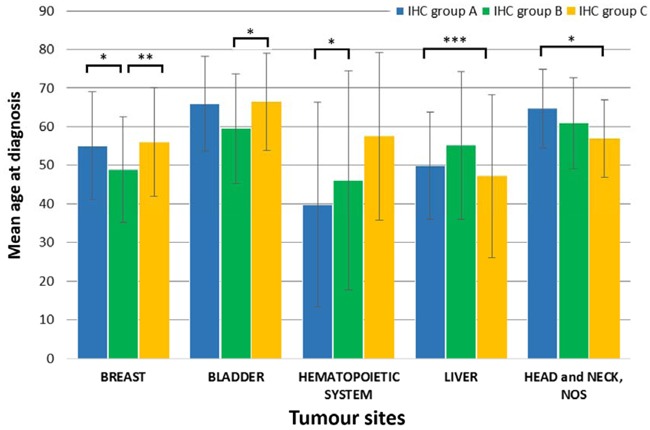
Significant differences of mean age of *TP53* mutation carriers between IHC groups were found in five tumour sites Bar graphs represent data in mean ± standard deviation (SD). Level of significance: * (*p* < 0.05), ** (*p* < 0.01), and *** (*p* < 0.001).

The majority of the mutations in hotspot p53 codons were strongly positive: 175 (89.7%), 245 (90.7%), 248 (93.3%), 249 (88.0%), 273 (92.6%) and 282 (90.9%). In contrast, mutations were usually negative among the frequently altered codons like 213 (57.7%), 196 (65.2%), 306 (60.9%), 146 (63.6%) and 298 (60.7%). More specifically, all alterations within the hotspot codons (R175H, G245S/D, R248Q/W/L, R249S, R273H/C/L, and R282W) were positive for p53 IHC at a range of 87.2-100%. Contrary, frequent nonsense mutations such as R213*, R196*, R306*, W146*, and E298* were not able to be detected (63.4-75.6%) by immunohistochemistry.

**Figure 3:**
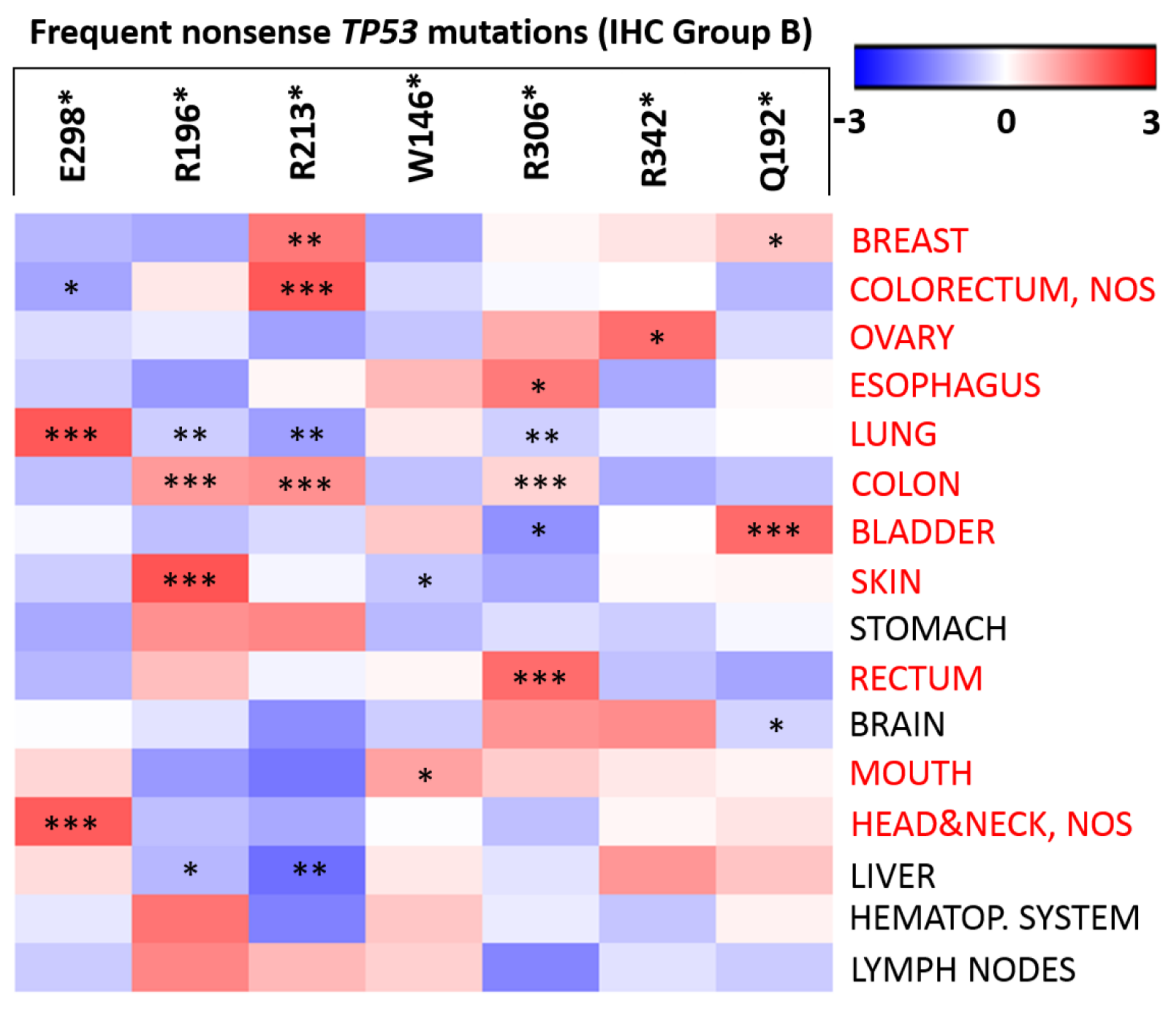
Frequency differences of nonsense *TP53* (Group B) mutations across tumour sites The ‘heat map’ shows the different frequency of the seven nonsense *TP53* mutations (ranging from −3 to 3) across tumour sites (compared to their overall frequency in IARC database). A heat-map of nonsense *TP53* mutations were constructed using Gene-E version 3.0.204 (http://www.broadinstitute.org/cancer/software/GENE-E/index.html). Level of significance: * (*p* < 0.05), ** (*p* < 0.01), and *** (*p* < 0.001).

Based on the mutation frequencies and IHC positivity, *TP53* mutations possess more than 15 IHC data (*n* = 78) were categorized into three distinct groups. Hot-spot and IHC positive mutations (9/78; 11.5%) were in Group A, frequent and mostly IHC negative mutations (7/78; 9%) were in Group B, whereas infrequent and IHC positive mutations were in Group C (62/78; 79.5%) (Figure 3). Principal components analysis of the groups by two components also confirmed differences between the three groups (Supplemental Figure S3). All alterations in Group A and C were missense, whereas in Group B they were nonsense (*p* < 0.001). The majority of *TP53* mutations in Group A and B localized within exons 5-8 (*p* < 0.001), and they commonly occurred in the CpG sequences (*p* < 0.001).

### Clinicopathological features of p53 IHC groups

Sex was available for 4897 *TP53* mutations with known IHC data, including 2797 females (57.2%) and 2097 males (42.9%). The p53 IHC was predominantly positive (75.8% *vs*. 76.4%) in both sex but females with IHC negative mutations were significantly younger (55.90 ± 15.391 *vs*. 58.57 ± 15.062 years, *p* = 0.0182). There was no significant difference regarding the mean age (56.44 ± 16.86 *vs*. 57.22 ± 15.772, *p* = 0.204). *TP53* alterations were IHC positive in almost all human cancers in the database at a range between 50 and 100%.

Sex distribution and the mean age at diagnosis were also evaluated in the three p53 IHC groups. A significant female dominance was observed in all groups (*p* = 0.0042). Significant differences were observed in five tumour sites (breast, bladder, haematopoietic system, liver, head and neck) in term of the age at diagnosis among the IHC groups (Figure 2). Breast cancer patients carrying nonsense mutations (Group B) were significantly younger (48.83±13.69 years) than patient in the other groups (Group A: 55.06±13.99 years & Group C: 55.97±14.07 years). Similar difference was observed in bladder cancer, but patients were notable younger in only Group B (59.52±14.20 years) compared to Group A (64.44±12.57 years). Contrary, liver carcinoma patients in Group A were significantly older then in Group C. Comparison of the Group A and C revealed that patients with frequent missense mutations were younger with haematopoietic system, but older with head and neck tumours. Mean ages in different IHC mutation groups are summarized in Supplemental Table S3.

Because the majority of nonsense *TP53* mutations were undetectable by IHC and carriers usually have worse overall survival and a poor prognosis, further characterisation of nonsense mutations in Group B was also important. Significant differences were observed between the total frequencies of the mutations in the IARC Database and their frequency in several tumour sites (Figure 3). For example, R213* was more common in breast, colorectum, colon, and less frequent in liver, lung compared to its overall frequency. Frequencies of nonsense mutations at various tumour sites are summarized in Supplemental Table S4.

## DISCUSSION

Despite the numerous studies, the association between *TP53* mutations and p53 nuclear accumulation is still not fully understood. Although immunohistochemistry is commonly used as a surrogate for *TP53* mutation analysis in the routine diagnostic work-up, its reliability is controversial due to the false-positive and negative cases. The aim of our study was to estimate the immunohistochemical expression patterns of *TP53* somatic mutations at various tumour sites and correlate them with clinicopathological features. Therefore, we performed a comprehensive evaluation of the R17 dataset from the IARC TP53 Database [1, 14].

In normal conditions, synthesis and degradation of p53 are strictly regulated, its expression level is maintained very low [15]. Aberrant p53 has a prolonged turnover resulting in an overexpressed protein that can be detected by IHC [16]. Our study confirm that more than 75% of *TP53* mutations showed IHC-detectable p53 accumulation in a unified manner. This absolute positivity was also observed in all tumour sites. This is in contrast with other tumour suppressor genes where mutations are mostly deletions or nonsense leading to a decreased or absent protein expression [3].

The majority of *TP53* mutations are predominantly clustered in the DNA binding domain (residues 94-292) [1]. In our results, over 94% of the IHC positive alterations also occurred in this domain (Supplemental Figure S2). The DNA-binding domain includes three particularly important regions. The L2 loop (codons 163-195) is required for the folding and stabilization of the central part of the protein, whereas L3 loop (codons 236-251) and loop-sheet-helix (L1/S/H2) motif (codons 273-286) can contact the DNA at least two residues (241, 248 and 273, 280, respectively). *TP53* mutations in these domains are associated with a more aggressive clinical phenotype, presumably they decrease biological activity of the protein [17, 18]. As indicated in Table 1, mutations within these crucial motifs are mostly positive by IHC.

The majority of *TP53* mutations occur within exons 5-8 [4]. In our study, more than 95% of the alterations localized within these exons and showed IHC positivity. Point mutations in *TP53* introns can abrogate the function of any remaining normal p53 [19]. Our findings are consistent with previous reports, because 87% of the IHC-detectable mutations were single base-pair substitutions in coding regions. These are missense mutations that usually produce full-length proteins, consequently, more than 80% of the alterations were also IHC positive according to the IARC database. Besides, the majority of tandem and complex *TP53* mutations are also IHC positive, however, they are relatively infrequent in human cancer [1]. Splice mutations in the *TP53* are reported as infrequent events. Accordingly, only the 3% of the mutations occurred within the conserved dinucleotides involved in splice sites and their distribution and IHC characteristics were diverse. The altered transcript is sufficiently stable and not degraded [20]. Splicing alterations in the alternative and cryptic splice acceptor sites showed IHC positivity; in contrast, they were negative in consensus splice sites. There was no remarkable difference in IHC expressions with respect to the position of the mutation within or outside CpG sequences, both of them were mostly positive (in 83.3% and 73.3%, respectively).

We also analysed the association between individual *TP53* mutations and their IHC characteristics. For this reason, somatic *TP53* mutations in the IARC database were classified into three groups based on their frequency and IHC positivity. The “hotspot” mutations (Group A) and frequent nonsense mutations (Group B) formed distinct groups, whereas the infrequent strongly positive missense mutations were in Group C (Figure 1). The key starting point of our study was that nonsense *TP53* mutations (Group B) result in lack of immunolabelling due to the absence of gene product [21]. Truncating mutations of genes like *NF2* or *HNF1A* are associated with an early age of onset [22, 23]. However, *TP53* mutations were predominantly IHC positive in both sexes. Females with IHC negative mutations (manly caused by truncating mutations) were younger. According to our classification of *TP53* mutations, the age at diagnosis of Group B was lower in breast and bladder cancers, and interestingly, it was higher in tumours of the haematopoietic system compared to Group A and C. The frequency of R213* and Q192* was also increased in breast, as well as R306* and Q192* mutations frequency in bladder cancer. In breast cancer, R213* *TP53* mutations are more frequent in the basal-like subtype [17]. In colon cancer, R196*, R213* and R306* mutations were also common. Furthermore, R213* mutation was common in colorectum, and R306* in rectum carcinomas. Vakiani *et al.* reported, that R306*mutation was present in 15.27% and 39.5% of alleles in the invasive component of the primary tumour and in the metastasis, respectively [18]. The W146* mutation was slightly more frequent in skin and mouth cancers in our analysis.

Although nonsense (Group B) mutations are infrequent, they indicate worse overall survival especially if p53 is truncated [24, 25]. Nadkarni *et al.* showed, that *TP53* null mutations presumably related to recurrent tumours. Their works indicate that deleterious alteration confers an increased and earlier probability for recurrence [26]. Furthermore, tumours with nonsense (Group B) mutations are more likely to develop metastatic tumours compared to those cancers that contain either missense mutations (Group A & C) or are wild-type p53 [27]. Moreover, patients harbouring null *TP53* alterations have an increased risk to have more vascular tumours [26]. The effect and importance of *TP53* mutations are summarized in a review paper by Muller and Vousden [28]. As anticipated, more than half of the alterations (56.5%) in the non-coding regions were mostly negative by IHC. We demonstrated that *TP53* mutations with negative IHC results were most frequently caused by deletions or insertions (Table 1). Drugs inducing the read-through of early stop codons caused by mutations could be a promising therapeutic strategy of the cancer linked with non-sense mutations in tumour suppressor genes. Aminoglycoside antibiotics such as gentamicin and G418 can promote premature termination codon read-through results in the partial restoration of full-length protein [29]. Floquet *et al*. described that Q192*, R213* and E298* *TP53* mutations displaying high induced read-through level [30]. The aminoglycoside treatment strongly and specifically stabilized mutant p53 mRNAs that would otherwise be degraded by non-sense mediated mRNA decay [30]. Although induction of read-through of premature stop codons is effective, the clinical use of these agents is still limited by their toxicity [31].

Currently, there is no consensus as to which antibodies are most appropriate for evaluating mutation-associated p53 expression [24]. None of the routinely used p53 antibodies (CM1, Pab1801, DO1 and DO7) differentiate between mutant and wild-type p53 proteins [2, 21]. While, CM1 antibody binds to the full length protein, DO7, DO1 and Pab1801 recognize epitopes only in the N-terminus of the human p53 protein (amino acid residues 1-45, 11-25 and 32-79, respectively) [32-34] (Supplemental Figure S2). Importantly, the most commonly used DO7 antibody can detect only truncation mutations in exons 9-10 [24]. A further limitation of p53 IHC is that not only *TP53* mutation but also disturbed p53 pathway can result in abnormal p53 expression [35]. Therefore, amplification of MDM2 or MDM4 as well as repressed p14^ARF^ or *TP53* by promoter methylation can cause reduced p53 expression resulting in a limited sensitivity of IHC [36, 37].

Intensive efforts have been made to improve the reliability of p53 IHC as a surrogate method. Combined usage of antibodies that target various p53 epitopes or p53-related proteins, as well as quantitative scoring methods seem to be valuable approaches for *TP53* mutation prediction. INenutil *et al.* applied a panel of eight antibodies that relate to p53 stabilization and transcriptional activation: anti-p53 (DO1; Bp53-10 & Pab1801; Ser15; Ser392), anti-Ki67 (MIB1), anti-MDM2 (2A9) and anti-p21 (118) [38]. They conclude that *i)* overexpressed p53 without increased MDM2 indicates inactivating mutations that stabilize p53; *ii)* tumours with overexpressed p53 and concurrent increase of MDM2 do not have p53 mutation. iii) phosphorylated p53 expression correlates with total p53 levels and iv) does not predict *TP53* mutation status [38]. Nevertheless there are no subsequent studies confirming these findings. Wertz *et al*. described, that a cocktail of DO1 and DO7 antibodies could identify 93% of cell lines and patient samples with *TP53* missense mutations in the exons 5 to 8 region in prostatic adenocarcinoma [39]. Combined IHC of PLK1 (Polo-like kinase-1), p21, and p53 is slightly more sensitive for predicting *TP53* status and may facilitate differentiation of missense and nonsense mutations [40]. The p21 is a transcriptional target of p53, therefore its expression is used for decreasing false positivity of p53 IHC. Immunohistochemical positivity of PLK1 along with negative p53 IHC can reflect nonsense *TP53* mutations and may decrease the possibility of false negative IHC, because mutant p53 fails to repress PLK1 expression [41]. Köbel *et al.* used p53 antibodies DO7, DO1, and E26 and tagged-amplicon next generation sequencing of *TP53* in high-grade serous ovarian carcinomas and endometrioid carcinomas. They demonstrated that optimized p53 IHC assay is a useful surrogate for the *TP53* mutation status, and that combination of p53 IHC and sequencing should be the gold standard in assessing the p53 functional status for clinical trial inclusion [42]. IHC scoring may correlate with *TP53* mutations and could increase the accuracy of p53 IHC. In a recent study, Cole *et al.* (2016) combined massively parallel sequencing and IHC to characterise *TP53* mutations and p53 expression in high-grade serous ovarian cancer. According to their results, missense *TP53* mutations have high expression of p53, whereas low expression was associated with non-missense mutations (i.e. frameshift, in-frame, nonsense, and splice). Furthermore, wild-type *TP53* tumours displayed intermediate p53 IHC expression [43].

A limitation of our analysis is that we cannot predict the overall, and tumour specific sensitivity of p53 immunohistochemistry because the IARC TP53 Database does not contain IHC results of wild-type p53 expression. Consequently, the analysis and exclusion of false positive cases has not been possible. IHC antibodies are not listed in the database, therefore their specificity and association with somatic mutations could not be analysed.

In summary, the majority of *TP53* mutations were missense and IHC positive, whereas most nonsense and frameshift mutations and deletions were immunonegative. Significant correlations were observed between the age at diagnosis and the immunohistochemical patterns of *TP53* mutations in breast, head & neck, bladder, liver and haematopoietic cancers. In certain tumour sites there is an increased likelihood of false negative IHC associated with rare nonsense mutations. Our frequency- and immunopositivity-based classification is useful in patient stratification and has prognostic implication.

**Table 2:** Comparison of p53 immunohistochemistry data with sex distribution and mean age of patients with *TP53* somatic mutations based on the IARC TP53 Database (R17)

	p53 IHC	Male	Female	*p*-value
Mean age at diagnosis (SD)	negative	58.567 (± 15.062)	55.896 (± 15.391)	0.0182^a^
	positive	57.220 (± 15.772)	56.439 (±16.860)	0.2646^a^
p53 IHC (%)	negative	496 (23.65%)	691 (24.71%)	0.8851^b^
	positive	1601 (76.35%)	2106 (75.29%)	

(IHC=immunohistochemistry; SD=Standard deviation;

a Mann-Witney U-test;

b Pearson's chi square test; Significant *p*-values are marked in bold)

## MATERIALS AND METHODS

### The IARC TP53 somatic mutation database

The last realised (R17) dataset of somatic *TP53* mutation (http://p53.iarc.fr/TP53SomaticMutations.aspx) was used for the characterisation of *TP53* somatic mutations, and correlate them with IHC expression data. Of the 29711 mutations, the protein description is available in 25361 mutations which are composed of 1778 unique *TP53* mutations. The nomenclature is at the protein level according to the Human Genome Variation Society (HGVS) standards, using the P04637 Uniprot reference sequence.

### Immunohistochemical characteristics of somatic *TP53* mutation

Somatic *TP53* mutations with known IHC data were retrieved and the expression patterns were analysed in various aspects, such as the localization within the *TP53* gene (exons/introns, CpG and splice sites); effects (frameshift, intronic, large deletion, missense, nonsense, silent, splice, other); type (single nucleotide mutations, tandem, complex, deletion, insertion); and affected structural motifs of p53 protein (C-terminal, C-terminal/NLS, C-terminal/tetramerization, L1/S/H2, L2/L3 loops, N-terminal, N-terminal/Transactivation, N-terminal/ Transactivation/ NES, NDBL/β-sheets, SH3-like/Proline-rich).

In the IARC database, p53 immunostaining is graded as ‘positive’ (*n* = 6026), ‘negative’ (*n* = 1852) and ‘*+/−*’ (*n* = 205). To improve the accuracy of our study only ‘positive’ and ‘negative’ p53 IHC results were considered. Based on these criteria, of the 29711 mutations IHC data are available for 7878 *TP53* somatic mutations corresponding to 1139 distinct alterations. *TP53* somatic mutations frequencies and their IHC data are summarized in Supplemental Table S1. The majority of the alterations (91.9%) are clustered within exons 5-8. More than two-thirds of the mutations (68.2%) occur in non-CpG sites, and 96.9% of the alterations are non-splice site mutations. Considering the mutation types, 6860 (87.1%) are single nucleotide substitutions (SNSs), 667 (8.5%) deletion, 196 (2.5%) insertion, 97 (1.2%) tandem and 48 (0.6%) complex mutation. Mutation type was non-available only in 10 mutations (0.1%). The most common mutation effect is missense (74.8%), followed by frameshift (8.6%), nonsense (7%), silent (4.7%), splicing (1.7%), intronic mutations (0.7%) and large deletion (0.03%). Further 2.3% of the mutations is specified as ‘other’ and 0.4% of the alterations remains undetermined (Table 1).

### Clinicopathological characteristics of *TP53* mutations with known IHC data

The subset of mutations with p53 IHC results compiles data on 7124 individuals. Sex is available for 4894 individuals (62.1%) including 2797 females (57.2%) and 2097 males (42.9%). The mean age at diagnosis is 56.90 ± 16.23 years given for 3072 patients (62.8%) (Table 2). The available p53 IHC expression data are distributed among 60 tumour sites (Supplemental Table S2). Information about the sample source is available for 6957 cases: surgery (83.3%), biopsy (13.5%), cell-line (2.7%), blood (0.3%), bone marrow (0.1%), xenograft (0.1%), pleural fluids (0.03%) and saliva (0.01%). Overall, 5211 mutations have a known origin: 4977 (95.5%) primary tumours, 106 (2%) metastasis, 84 (1.6%) recurrent, and 44 (0.8%) secondary tumours.

### Classification of *TP53* mutations based on frequency and IHC patterns

We investigated the frequency of 1139 distinct *TP53* mutations in the IARC Database and IHC positivity of individual mutations was also calculated by dividing the IHC positive cases by the total number of the given mutation (Supplemental Table S1). Based on these characteristics, we divided *TP53* somatic mutations into three groups: 1.) Group A (strongly IHC positive, hot-spot mutations; mutation frequency ≥ 0.013 and IHC positivity ≥ 0.85), 2.) Group B (mainly IHC negative nonsense mutations; mutation frequency ≤ 0.011 and IHC positivity ≤ 0.44) and 3.) Group C (IHC positive, non-frequent missense mutations; mutation frequency ≤ 0.072 and IHC positivity ≥ 0.74). Only individual mutations with 15 or more IHC results were involved in the classification. To evaluate these two components we applied principal component analysis (PCA).

### Statistical analysis

Dataset of *TP53* somatic mutations was downloaded and extracted using Microsoft Excel version 2013 (Microsoft Corp, Redmond, Washington, USA) and the statistical analysis was performed using SPSS 19.0 (SPSS Inc., Chicago IL) for Windows and R statistical software (www.R-project.org). Comparisons between groups were performed with Pearson's Chi-square (χ2) test for categorical variables. Age of onset associations between groups were done with the Mann-Whitney U test. Two-sided tests were computed and statistical significance was set at *p* < 0.05. The difference in proportions was compared using 2-sample test for equality of proportions with continuity correction.

## SUPPLEMENTARY MATERIAL FIGURES AND TABLES




