# Supplementary material for: Immunohistochemical correlates of TP53 somatic mutations in cancer
Source: Oncotarget. 2016 Sep 8;7(40):64910–20. doi: 10.18632/oncotarget.11912 (PMC5323125; doi:10.18632/oncotarget.11912)
Supplement: Supplementary file 1 [file oncotarget-07-64910-s001.pdf]

(B).

Immunohistochemical correlates of *TP53* somatic mutations in cancer

Supplementary Material

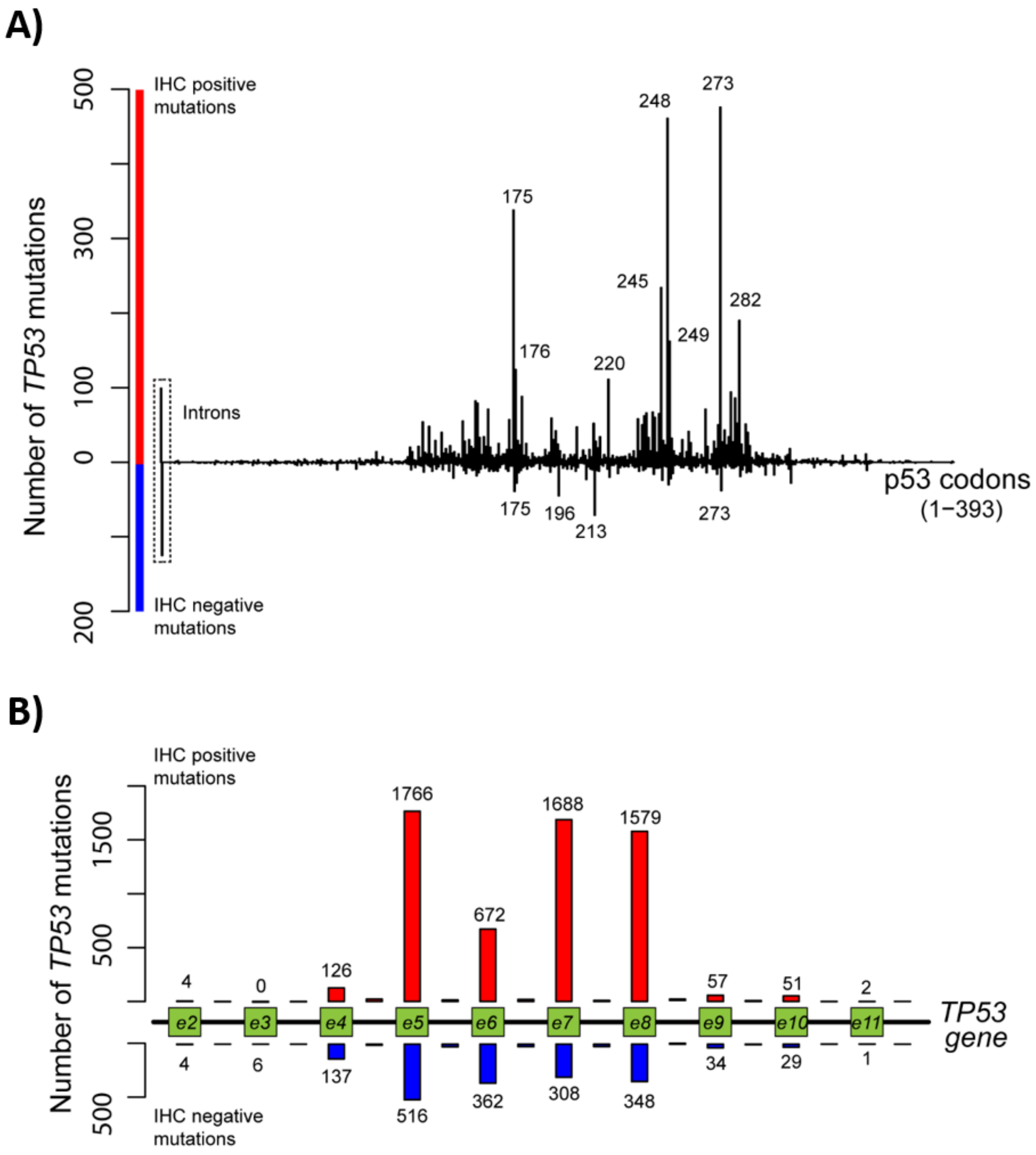

Figure S1. Immunohistochemical characteristics of p53 codons (A) and *TP53* exons/introns (B).

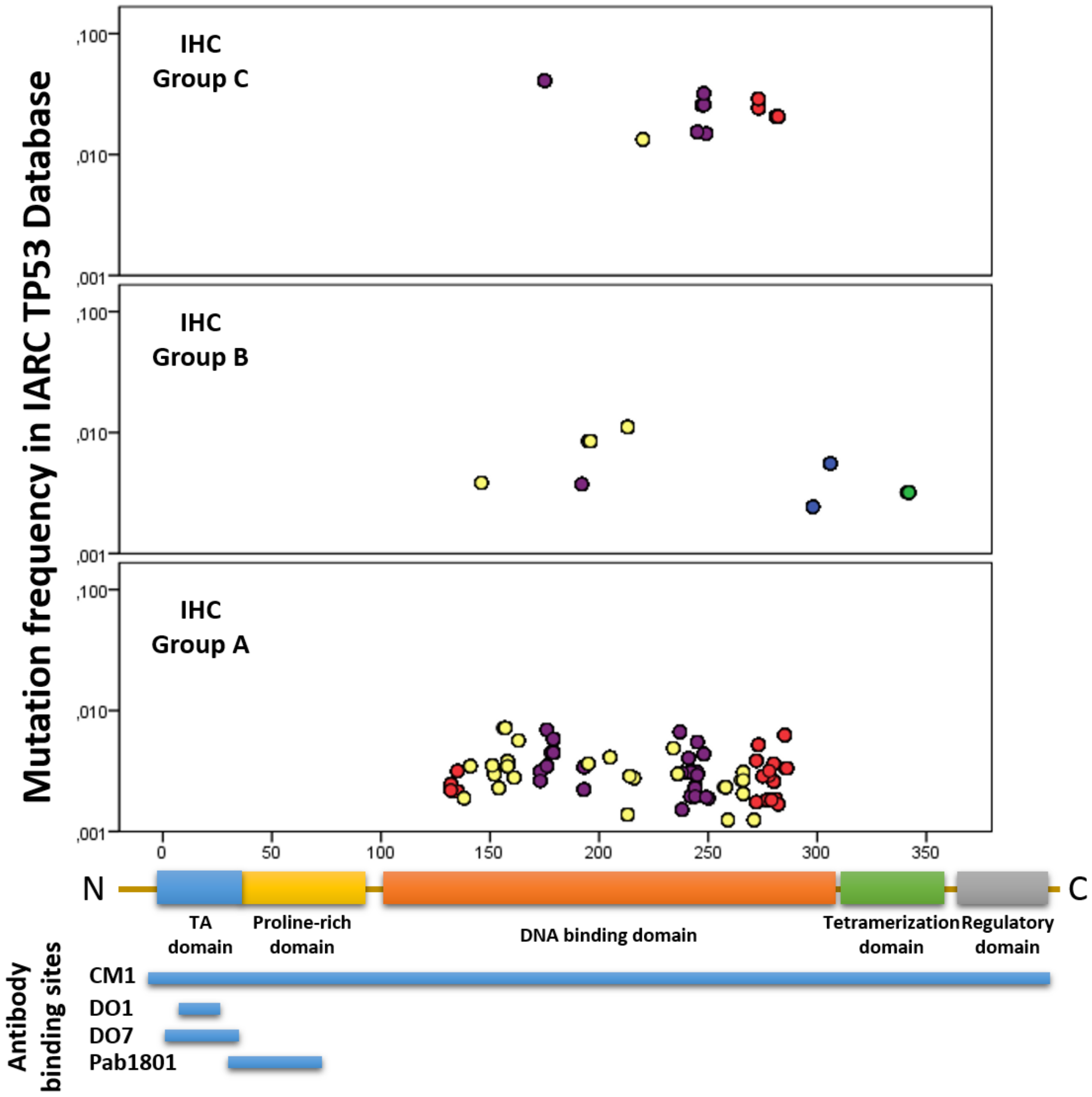

Figure S2. Schematic representation of the human p53 protein with the mutations of each IHC groups and the binding sites of the commonly used p53 IHC antibodies. Individual mutations are marked with circles; different colours represent p53 structural motifs (blue= C-terminal region; green=C-terminal/tetramerization domain; red=L1/S/H2; purple=L2/L3; yellow=NDBL/Beta-sheets).

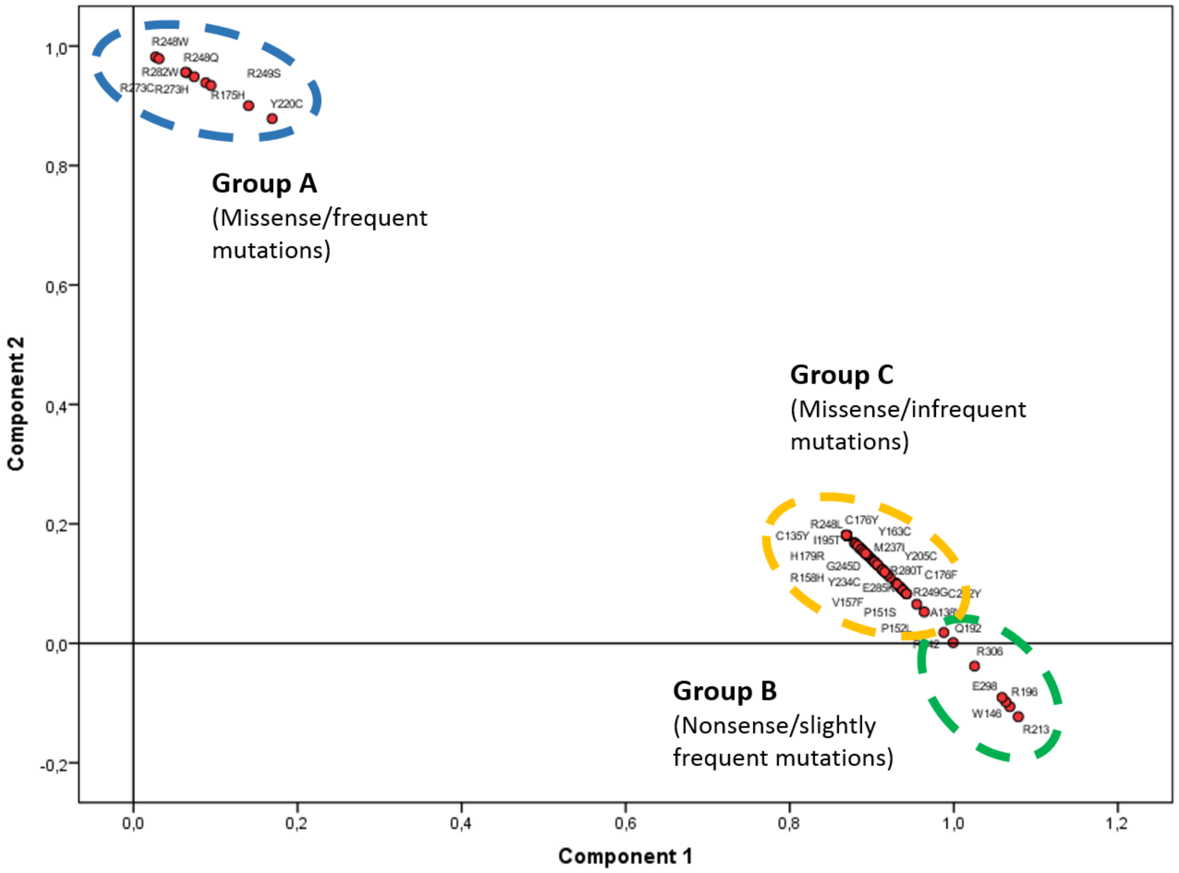

Figure S3. Principal components analysis (PCA) of the three IHC groups by two components.

Table S1. *Summary of individual TP53 somatic mutations with known IHC data*

Table S2. *Distribution of p53 IHC expression data among tumour sites in the IARC TP53 Database*

Table S3. *Comparison of mean ages of patient carrying TP53 mtations among different IHC groups*

Table S4. *Summary of nonsense mutation frequencies in different tumour sites*

For Table S1, S2, S3, S4 please see the attached word file
