# Supplementary material for: Immunohistochemical correlates of TP53 somatic mutations in cancer
Source: Oncotarget. 2016 Sep 8;7(40):64910–20. doi: 10.18632/oncotarget.11912 (PMC5323125; doi:10.18632/oncotarget.11912)
Supplement: Supplementary file 2 [file oncotarget-07-64910-s002.docx]

**Immunohistochemical correlates of *TP53* somatic mutations in cancer**

**Balázs Murnyák^1^, Tibor Hortobágyi^1,2*^**

**Supplementary Material**

| **Table S1.** *Summary of individual TP53 somatic mutations with known IHC data* | | | | | | |  |
| --- | --- | --- | --- | --- | --- | --- | --- |
| **Somatic *TP53* mutations** | **n** | **p53 IHC available** | **Negative IHC Data** | **Positive IHC Data** | **Mutation frequency in IARC** | **p53 IHC positivity** |  |
| **R175H** | 1215 | 336 | 29 | 307 | 0.040893945 | 0.913690476 | **Mutations were involved in the three IHC groups** |
| **R248Q** | 949 | 231 | 16 | 215 | 0.031941032 | 0.930735931 |  |
| **R248W** | 765 | 226 | 9 | 217 | 0.025748039 | 0.960176991 |  |
| **R273H** | 856 | 222 | 17 | 205 | 0.028810878 | 0.923423423 |  |
| **R273C** | 717 | 208 | 9 | 199 | 0.024132476 | 0.956730769 |  |
| **R282W** | 613 | 174 | 12 | 162 | 0.020632089 | 0.931034483 |  |
| **G245S** | 457 | 139 | 13 | 126 | 0.015381509 | 0.90647482 |  |
| **Y220C** | 396 | 120 | 18 | 102 | 0.013328397 | 0.85 |  |
| **R249S** | 442 | 109 | 14 | 95 | 0.014876645 | 0.871559633 |  |
| **R213*** | 330 | 90 | 68 | 22 | 0.011106997 | 0.244444444 |  |
| **C176F** | 206 | 83 | 15 | 68 | 0.006933459 | 0.819277108 |  |
| **V157F** | 213 | 58 | 10 | 48 | 0.007169062 | 0.827586207 |  |
| **M237I** | 198 | 58 | 5 | 53 | 0.006664198 | 0.913793103 |  |
| **R196*** | 252 | 56 | 41 | 15 | 0.008481707 | 0.267857143 |  |
| **Y163C** | 168 | 55 | 3 | 52 | 0.005654471 | 0.945454545 |  |
| **G245D** | 163 | 50 | 3 | 47 | 0.005486184 | 0.94 |  |
| **R273L** | 155 | 47 | 2 | 45 | 0.005216923 | 0.957446809 |  |
| **E285K** | 186 | 46 | 8 | 38 | 0.006260308 | 0.826086957 |  |
| **S241F** | 120 | 42 | 1 | 41 | 0.004038908 | 0.976190476 |  |
| **R306*** | 165 | 41 | 26 | 15 | 0.005553499 | 0.365853659 |  |
| **Y234C** | 145 | 41 | 5 | 36 | 0.004880347 | 0.87804878 |  |
| **H179R** | 173 | 39 | 1 | 38 | 0.005822759 | 0.974358974 |  |
| **Y205C** | 122 | 36 | 4 | 32 | 0.004106223 | 0.888888889 |  |
| **P278L** | 86 | 35 | 1 | 34 | 0.002894551 | 0.971428571 |  |
| **G266E** | 92 | 34 | 2 | 32 | 0.003096496 | 0.941176471 |  |
| **V272M** | 114 | 32 | 3 | 29 | 0.003836963 | 0.90625 |  |
| **C141Y** | 103 | 31 | 5 | 26 | 0.003466729 | 0.838709677 |  |
| **H179Y** | 133 | 30 | 2 | 28 | 0.004476457 | 0.933333333 |  |
| **R158H** | 113 | 30 | 3 | 27 | 0.003803305 | 0.9 |  |
| **R158L** | 103 | 30 | 3 | 27 | 0.003466729 | 0.9 |  |
| **H214R** | 85 | 30 | 5 | 25 | 0.002860893 | 0.833333333 |  |
| **W146*** | 114 | 29 | 21 | 8 | 0.003836963 | 0.275862069 |  |
| **P151S** | 104 | 28 | 5 | 23 | 0.003500387 | 0.821428571 |  |
| **R280K** | 77 | 28 | 1 | 27 | 0.002591633 | 0.964285714 |  |
| **C238Y** | 91 | 27 | 2 | 25 | 0.003062839 | 0.925925926 |  |
| **V173L** | 93 | 26 | 0 | 26 | 0.003130154 | 1 |  |
| **R280T** | 108 | 25 | 3 | 22 | 0.003635017 | 0.88 |  |
| **E286K** | 99 | 25 | 1 | 24 | 0.003332099 | 0.96 |  |
| **G245C** | 92 | 25 | 2 | 23 | 0.003096496 | 0.92 |  |
| **P278S** | 94 | 24 | 1 | 23 | 0.003163811 | 0.958333333 |  |
| **V173M** | 78 | 24 | 0 | 24 | 0.00262529 | 1 |  |
| **V216M** | 82 | 23 | 2 | 21 | 0.002759921 | 0.913043478 |  |
| **I195T** | 108 | 22 | 0 | 22 | 0.003635017 | 1 |  |
| **C176Y** | 103 | 22 | 0 | 22 | 0.003466729 | 1 |  |
| **C242F** | 92 | 22 | 0 | 22 | 0.003096496 | 1 |  |
| **Y236C** | 89 | 22 | 2 | 20 | 0.002995524 | 0.909090909 |  |
| **G245V** | 87 | 22 | 2 | 20 | 0.002928208 | 0.909090909 |  |
| **G266R** | 79 | 22 | 2 | 20 | 0.002658948 | 0.909090909 |  |
| **H193R** | 101 | 21 | 1 | 20 | 0.003399414 | 0.952380952 |  |
| **C275Y** | 85 | 21 | 4 | 17 | 0.002860893 | 0.80952381 |  |
| **K132N** | 73 | 21 | 3 | 18 | 0.002457002 | 0.857142857 |  |
| **E298*** | 72 | 21 | 15 | 6 | 0.002423345 | 0.285714286 |  |
| **E258K** | 69 | 21 | 1 | 20 | 0.002322372 | 0.952380952 |  |
| **A161T** | 83 | 20 | 1 | 19 | 0.002793578 | 0.95 |  |
| **G244S** | 70 | 20 | 0 | 20 | 0.00235603 | 1 |  |
| **C242Y** | 58 | 20 | 4 | 16 | 0.001952139 | 0.8 |  |
| **P250L** | 56 | 20 | 2 | 18 | 0.001884824 | 0.9 |  |
| **Q192*** | 111 | 19 | 11 | 8 | 0.00373599 | 0.421052632 |  |
| **A138V** | 56 | 19 | 5 | 14 | 0.001884824 | 0.736842105 |  |
| **R248L** | 130 | 18 | 0 | 18 | 0.004375484 | 1 |  |
| **R342*** | 95 | 18 | 10 | 8 | 0.003197469 | 0.444444444 |  |
| **C135Y** | 94 | 18 | 0 | 18 | 0.003163811 | 1 |  |
| **G244C** | 58 | 18 | 1 | 17 | 0.001952139 | 0.944444444 |  |
| **P152L** | 88 | 17 | 4 | 13 | 0.002961866 | 0.764705882 |  |
| **G266V** | 61 | 17 | 2 | 15 | 0.002053112 | 0.882352941 |  |
| **G279E** | 54 | 17 | 2 | 15 | 0.001817509 | 0.882352941 |  |
| **C277F** | 54 | 17 | 2 | 15 | 0.001817509 | 0.882352941 |  |
| **G244D** | 68 | 16 | 1 | 15 | 0.002288715 | 0.9375 |  |
| **G154V** | 68 | 16 | 0 | 16 | 0.002288715 | 1 |  |
| **K132R** | 65 | 16 | 3 | 13 | 0.002187742 | 0.8125 |  |
| **V272L** | 52 | 16 | 0 | 16 | 0.001750194 | 1 |  |
| **R282G** | 50 | 16 | 2 | 14 | 0.001682878 | 0.875 |  |
| **R213L** | 41 | 16 | 1 | 15 | 0.00137996 | 0.9375 |  |
| **D259Y** | 37 | 16 | 1 | 15 | 0.00124533 | 0.9375 |  |
| **H193L** | 66 | 15 | 1 | 14 | 0.002221399 | 0.933333333 |  |
| **C135F** | 64 | 15 | 1 | 14 | 0.002154084 | 0.933333333 |  |
| **R249G** | 57 | 15 | 3 | 12 | 0.001918481 | 0.8 |  |
| **D281E** | 55 | 15 | 2 | 13 | 0.001851166 | 0.866666667 |  |
| **C238F** | 45 | 15 | 3 | 12 | 0.001514591 | 0.8 |  |
| E271K | 37 | 15 | 5 | 10 | 0.00124533 | 0.666666667 |  |
| M246V | 61 | 14 | 1 | 13 | 0.002053112 | 0.928571429 |  |
| N239D | 52 | 14 | 3 | 11 | 0.001750194 | 0.785714286 |  |
| R249W | 50 | 14 | 2 | 12 | 0.001682878 | 0.857142857 |  |
| R280G | 46 | 14 | 1 | 13 | 0.001548248 | 0.928571429 |  |
| R273P | 38 | 14 | 0 | 14 | 0.001278988 | 1 |  |
| R249M | 70 | 13 | 0 | 13 | 0.00235603 | 1 |  |
| L194R | 68 | 13 | 1 | 12 | 0.002288715 | 0.923076923 |  |
| E294* | 65 | 13 | 11 | 2 | 0.002187742 | 0.153846154 |  |
| A159V | 57 | 13 | 2 | 11 | 0.001918481 | 0.846153846 |  |
| I255F | 42 | 13 | 1 | 12 | 0.001413618 | 0.923076923 |  |
| D281H | 42 | 13 | 0 | 13 | 0.001413618 | 1 |  |
| F270L | 33 | 13 | 3 | 10 | 0.0011107 | 0.769230769 |  |
| C238S | 32 | 13 | 1 | 12 | 0.001077042 | 0.923076923 |  |
| C275F | 54 | 12 | 0 | 12 | 0.001817509 | 1 |  |
| H179L | 47 | 12 | 0 | 12 | 0.001581906 | 1 |  |
| P177L | 38 | 12 | 5 | 7 | 0.001278988 | 0.583333333 |  |
| I195F | 34 | 12 | 1 | 11 | 0.001144357 | 0.916666667 |  |
| R280I | 26 | 12 | 0 | 12 | 0.000875097 | 1 |  |
| H193Y | 43 | 11 | 1 | 10 | 0.001447275 | 0.909090909 |  |
| P151H | 42 | 11 | 0 | 11 | 0.001413618 | 1 |  |
| D281N | 37 | 11 | 0 | 11 | 0.00124533 | 1 |  |
| S241C | 35 | 11 | 2 | 9 | 0.001178015 | 0.818181818 |  |
| C176S | 35 | 11 | 0 | 11 | 0.001178015 | 1 |  |
| R249K | 34 | 11 | 0 | 11 | 0.001144357 | 1 |  |
| A138P | 33 | 11 | 0 | 11 | 0.0011107 | 1 |  |
| T155N | 32 | 11 | 1 | 10 | 0.001077042 | 0.909090909 |  |
| R181C | 32 | 11 | 2 | 9 | 0.001077042 | 0.818181818 |  |
| R158C | 31 | 11 | 1 | 10 | 0.001043385 | 0.909090909 |  |
| R175C | 29 | 11 | 3 | 8 | 0.000976069 | 0.727272727 |  |
| C277Y | 28 | 11 | 0 | 11 | 0.000942412 | 1 |  |
| E204* | 51 | 10 | 7 | 3 | 0.001716536 | 0.3 |  |
| Q165* | 50 | 10 | 9 | 1 | 0.001682878 | 0.1 |  |
| P278R | 44 | 10 | 0 | 10 | 0.001480933 | 1 |  |
| V274F | 34 | 10 | 0 | 10 | 0.001144357 | 1 |  |
| P278T | 34 | 10 | 0 | 10 | 0.001144357 | 1 |  |
| R267W | 33 | 10 | 1 | 9 | 0.0011107 | 0.9 |  |
| R249T | 32 | 10 | 0 | 10 | 0.001077042 | 1 |  |
| E258* | 27 | 10 | 6 | 4 | 0.000908754 | 0.4 |  |
| K164E | 25 | 10 | 1 | 9 | 0.000841439 | 0.9 |  |
| L145P | 24 | 10 | 1 | 9 | 0.000807782 | 0.9 |  |
| V197G | 22 | 10 | 2 | 8 | 0.000740466 | 0.8 |  |
| Y236N | 21 | 10 | 0 | 10 | 0.000706809 | 1 |  |
| T211I | 20 | 10 | 2 | 8 | 0.000673151 | 0.8 |  |
| K305* | 20 | 10 | 5 | 5 | 0.000673151 | 0.5 |  |
| V157I | 19 | 10 | 2 | 8 | 0.000639494 | 0.8 |  |
| R156P | 45 | 9 | 2 | 7 | 0.001514591 | 0.777777778 |  |
| R213Q | 40 | 9 | 0 | 9 | 0.001346303 | 1 |  |
| S127F | 37 | 9 | 1 | 8 | 0.00124533 | 0.888888889 |  |
| R283P | 36 | 9 | 1 | 8 | 0.001211672 | 0.888888889 |  |
| R181H | 33 | 9 | 5 | 4 | 0.0011107 | 0.444444444 |  |
| R280S | 29 | 9 | 0 | 9 | 0.000976069 | 1 |  |
| L194F | 29 | 9 | 1 | 8 | 0.000976069 | 0.888888889 |  |
| F134L | 29 | 9 | 2 | 7 | 0.000976069 | 0.777777778 |  |
| W91* | 26 | 9 | 9 | 0 | 0.000875097 | 0 |  |
| E286* | 24 | 9 | 8 | 1 | 0.000807782 | 0.111111111 |  |
| Y236* | 23 | 9 | 6 | 3 | 0.000774124 | 0.333333333 |  |
| R158P | 23 | 9 | 1 | 8 | 0.000774124 | 0.888888889 |  |
| E286G | 22 | 9 | 2 | 7 | 0.000740466 | 0.777777778 |  |
| R273S | 21 | 9 | 3 | 6 | 0.000706809 | 0.666666667 |  |
| P177P | 20 | 9 | 5 | 4 | 0.000673151 | 0.444444444 |  |
| G154G | 20 | 9 | 4 | 5 | 0.000673151 | 0.555555556 |  |
| K132Q | 19 | 9 | 1 | 8 | 0.000639494 | 0.888888889 |  |
| Q100* | 18 | 9 | 8 | 1 | 0.000605836 | 0.111111111 |  |
| Q136* | 53 | 8 | 7 | 1 | 0.001783851 | 0.125 |  |
| P190L | 51 | 8 | 1 | 7 | 0.001716536 | 0.875 |  |
| M246I | 48 | 8 | 2 | 6 | 0.001615563 | 0.75 |  |
| C242S | 36 | 8 | 0 | 8 | 0.001211672 | 1 |  |
| S166* | 34 | 8 | 7 | 1 | 0.001144357 | 0.125 |  |
| S215R | 31 | 8 | 3 | 5 | 0.001043385 | 0.625 |  |
| Y126* | 30 | 8 | 5 | 3 | 0.001009727 | 0.375 |  |
| R175L | 30 | 8 | 2 | 6 | 0.001009727 | 0.75 |  |
| Y163H | 29 | 8 | 1 | 7 | 0.000976069 | 0.875 |  |
| F270C | 27 | 8 | 2 | 6 | 0.000908754 | 0.75 |  |
| K132E | 25 | 8 | 0 | 8 | 0.000841439 | 1 |  |
| C238R | 24 | 8 | 0 | 8 | 0.000807782 | 1 |  |
| D208V | 19 | 8 | 1 | 7 | 0.000639494 | 0.875 |  |
| V172I | 13 | 8 | 3 | 5 | 0.000437548 | 0.625 |  |
| I162I | 10 | 8 | 2 | 6 | 0.000336576 | 0.75 |  |
| T211T | 9 | 8 | 2 | 6 | 0.000302918 | 0.75 |  |
| Q144* | 48 | 7 | 4 | 3 | 0.001615563 | 0.428571429 |  |
| Q167* | 44 | 7 | 5 | 2 | 0.001480933 | 0.285714286 |  |
| S183* | 42 | 7 | 4 | 3 | 0.001413618 | 0.428571429 |  |
| Q331* | 32 | 7 | 4 | 3 | 0.001077042 | 0.428571429 |  |
| D184N | 32 | 7 | 1 | 6 | 0.001077042 | 0.857142857 |  |
| R282Q | 30 | 7 | 1 | 6 | 0.001009727 | 0.857142857 |  |
| P278A | 30 | 7 | 2 | 5 | 0.001009727 | 0.714285714 |  |
| G266* | 29 | 7 | 5 | 2 | 0.000976069 | 0.285714286 |  |
| R248G | 27 | 7 | 0 | 7 | 0.000908754 | 1 |  |
| T155I | 26 | 7 | 1 | 6 | 0.000875097 | 0.857142857 |  |
| Y163N | 25 | 7 | 0 | 7 | 0.000841439 | 1 |  |
| R181P | 24 | 7 | 0 | 7 | 0.000807782 | 1 |  |
| R175G | 24 | 7 | 1 | 6 | 0.000807782 | 0.857142857 |  |
| H168R | 24 | 7 | 1 | 6 | 0.000807782 | 0.857142857 |  |
| R158G | 23 | 7 | 0 | 7 | 0.000774124 | 1 |  |
| A161D | 23 | 7 | 0 | 7 | 0.000774124 | 1 |  |
| C176W | 21 | 7 | 0 | 7 | 0.000706809 | 1 |  |
| Y163* | 19 | 7 | 5 | 2 | 0.000639494 | 0.285714286 |  |
| N247N | 17 | 7 | 2 | 5 | 0.000572179 | 0.714285714 |  |
| E286Q | 16 | 7 | 1 | 6 | 0.000538521 | 0.857142857 |  |
| P278H | 15 | 7 | 0 | 7 | 0.000504864 | 1 |  |
| R110C | 12 | 7 | 1 | 6 | 0.000403891 | 0.857142857 |  |
| R174S | 10 | 7 | 1 | 6 | 0.000336576 | 0.857142857 |  |
| V143M | 35 | 6 | 0 | 6 | 0.001178015 | 1 |  |
| R290H | 34 | 6 | 0 | 6 | 0.001144357 | 1 |  |
| P152S | 34 | 6 | 1 | 5 | 0.001144357 | 0.833333333 |  |
| Y234H | 33 | 6 | 1 | 5 | 0.0011107 | 0.833333333 |  |
| H179Q | 30 | 6 | 0 | 6 | 0.001009727 | 1 |  |
| S215I | 27 | 6 | 0 | 6 | 0.000908754 | 1 |  |
| E198* | 27 | 6 | 6 | 0 | 0.000908754 | 0 |  |
| K164* | 25 | 6 | 5 | 1 | 0.000841439 | 0.166666667 |  |
| H179N | 25 | 6 | 1 | 5 | 0.000841439 | 0.833333333 |  |
| H179D | 24 | 6 | 2 | 4 | 0.000807782 | 0.666666667 |  |
| G244V | 24 | 6 | 1 | 5 | 0.000807782 | 0.833333333 |  |
| E171* | 24 | 6 | 4 | 2 | 0.000807782 | 0.333333333 |  |
| Y126C | 21 | 6 | 1 | 5 | 0.000706809 | 0.833333333 |  |
| V274A | 21 | 6 | 1 | 5 | 0.000706809 | 0.833333333 |  |
| P153S | 21 | 6 | 2 | 4 | 0.000706809 | 0.666666667 |  |
| E271* | 21 | 6 | 5 | 1 | 0.000706809 | 0.166666667 |  |
| E258G | 21 | 6 | 1 | 5 | 0.000706809 | 0.833333333 |  |
| E180* | 21 | 6 | 4 | 2 | 0.000706809 | 0.333333333 |  |
| D259V | 21 | 6 | 1 | 5 | 0.000706809 | 0.833333333 |  |
| R267P | 20 | 6 | 1 | 5 | 0.000673151 | 0.833333333 |  |
| P151A | 20 | 6 | 0 | 6 | 0.000673151 | 1 |  |
| L145Q | 20 | 6 | 0 | 6 | 0.000673151 | 1 |  |
| D259N | 20 | 6 | 4 | 2 | 0.000673151 | 0.333333333 |  |
| R282R | 19 | 6 | 1 | 5 | 0.000639494 | 0.833333333 |  |
| R273G | 19 | 6 | 1 | 5 | 0.000639494 | 0.833333333 |  |
| P177S | 19 | 6 | 0 | 6 | 0.000639494 | 1 |  |
| H193P | 19 | 6 | 0 | 6 | 0.000639494 | 1 |  |
| G245R | 19 | 6 | 0 | 6 | 0.000639494 | 1 |  |
| A276P | 19 | 6 | 0 | 6 | 0.000639494 | 1 |  |
| R283H | 18 | 6 | 1 | 5 | 0.000605836 | 0.833333333 |  |
| R209* | 18 | 6 | 6 | 0 | 0.000605836 | 0 |  |
| G187S | 18 | 6 | 4 | 2 | 0.000605836 | 0.333333333 |  |
| I255T | 17 | 6 | 0 | 6 | 0.000572179 | 1 |  |
| Y234N | 16 | 6 | 1 | 5 | 0.000538521 | 0.833333333 |  |
| M169I | 16 | 6 | 0 | 6 | 0.000538521 | 1 |  |
| G154S | 15 | 6 | 2 | 4 | 0.000504864 | 0.666666667 |  |
| A276D | 14 | 6 | 0 | 6 | 0.000471206 | 1 |  |
| L130R | 11 | 6 | 0 | 6 | 0.000370233 | 1 |  |
| P300L | 10 | 6 | 2 | 4 | 0.000336576 | 0.666666667 |  |
| V147G | 9 | 6 | 0 | 6 | 0.000302918 | 1 |  |
| T102I | 8 | 6 | 1 | 5 | 0.000269261 | 0.833333333 |  |
| L264L | 6 | 6 | 4 | 2 | 0.000201945 | 0.333333333 |  |
| Q317* | 40 | 5 | 4 | 1 | 0.001346303 | 0.2 |  |
| A159P | 33 | 5 | 0 | 5 | 0.0011107 | 1 |  |
| R283C | 29 | 5 | 1 | 4 | 0.000976069 | 0.8 |  |
| C135W | 28 | 5 | 0 | 5 | 0.000942412 | 1 |  |
| L130V | 23 | 5 | 1 | 4 | 0.000774124 | 0.8 |  |
| R282P | 22 | 5 | 0 | 5 | 0.000740466 | 1 |  |
| R248R | 22 | 5 | 1 | 4 | 0.000740466 | 0.8 |  |
| P151T | 22 | 5 | 1 | 4 | 0.000740466 | 0.8 |  |
| V143A | 21 | 5 | 0 | 5 | 0.000706809 | 1 |  |
| Y205S | 20 | 5 | 0 | 5 | 0.000673151 | 1 |  |
| V172F | 20 | 5 | 0 | 5 | 0.000673151 | 1 |  |
| R196P | 20 | 5 | 0 | 5 | 0.000673151 | 1 |  |
| P250S | 20 | 5 | 0 | 5 | 0.000673151 | 1 |  |
| I195N | 20 | 5 | 0 | 5 | 0.000673151 | 1 |  |
| E224D | 20 | 5 | 3 | 2 | 0.000673151 | 0.4 |  |
| P177R | 19 | 5 | 2 | 3 | 0.000639494 | 0.6 |  |
| A159A | 19 | 5 | 3 | 2 | 0.000639494 | 0.4 |  |
| S240R | 18 | 5 | 2 | 3 | 0.000605836 | 0.6 |  |
| P151P | 18 | 5 | 1 | 4 | 0.000605836 | 0.8 |  |
| C141R | 18 | 5 | 0 | 5 | 0.000605836 | 1 |  |
| R267Q | 17 | 5 | 0 | 5 | 0.000572179 | 1 |  |
| R174W | 16 | 5 | 1 | 4 | 0.000538521 | 0.8 |  |
| E287* | 16 | 5 | 3 | 2 | 0.000538521 | 0.4 |  |
| Y126_K132del | 15 | 5 | 2 | 3 | 0.000504864 | 0.6 |  |
| V216L | 15 | 5 | 0 | 5 | 0.000504864 | 1 |  |
| M243I | 15 | 5 | 3 | 2 | 0.000504864 | 0.4 |  |
| L194P | 15 | 5 | 1 | 4 | 0.000504864 | 0.8 |  |
| V157G | 14 | 5 | 0 | 5 | 0.000471206 | 1 |  |
| H168P | 14 | 5 | 0 | 5 | 0.000471206 | 1 |  |
| F270I | 14 | 5 | 2 | 3 | 0.000471206 | 0.6 |  |
| C242R | 14 | 5 | 1 | 4 | 0.000471206 | 0.8 |  |
| C141W | 14 | 5 | 2 | 3 | 0.000471206 | 0.6 |  |
| A159D | 14 | 5 | 0 | 5 | 0.000471206 | 1 |  |
| S227F | 13 | 5 | 1 | 4 | 0.000437548 | 0.8 |  |
| E180K | 13 | 5 | 0 | 5 | 0.000437548 | 1 |  |
| V172V | 12 | 5 | 3 | 2 | 0.000403891 | 0.4 |  |
| T170T | 12 | 5 | 2 | 3 | 0.000403891 | 0.6 |  |
| R290C | 12 | 5 | 1 | 4 | 0.000403891 | 0.8 |  |
| H178P | 12 | 5 | 1 | 4 | 0.000403891 | 0.8 |  |
| E224* | 12 | 5 | 5 | 0 | 0.000403891 | 0 |  |
| A161A | 12 | 5 | 1 | 4 | 0.000403891 | 0.8 |  |
| V274D | 10 | 5 | 0 | 5 | 0.000336576 | 1 |  |
| G245L | 10 | 5 | 1 | 4 | 0.000336576 | 0.8 |  |
| V157L | 9 | 5 | 1 | 4 | 0.000302918 | 0.8 |  |
| T231T | 9 | 5 | 2 | 3 | 0.000302918 | 0.6 |  |
| N268S | 9 | 5 | 3 | 2 | 0.000302918 | 0.4 |  |
| H168L | 9 | 5 | 1 | 4 | 0.000302918 | 0.8 |  |
| Y234S | 8 | 5 | 1 | 4 | 0.000269261 | 0.8 |  |
| E287E | 8 | 5 | 1 | 4 | 0.000269261 | 0.8 |  |
| A159T | 8 | 5 | 0 | 5 | 0.000269261 | 1 |  |
| S261R | 7 | 5 | 0 | 5 | 0.000235603 | 1 |  |
| H178N | 7 | 5 | 0 | 5 | 0.000235603 | 1 |  |
| R110L | 36 | 4 | 0 | 4 | 0.001211672 | 1 |  |
| N239S | 35 | 4 | 0 | 4 | 0.001178015 | 1 |  |
| I251S | 26 | 4 | 0 | 4 | 0.000875097 | 1 |  |
| S241Y | 23 | 4 | 0 | 4 | 0.000774124 | 1 |  |
| N235S | 22 | 4 | 0 | 4 | 0.000740466 | 1 |  |
| L257Q | 21 | 4 | 0 | 4 | 0.000706809 | 1 |  |
| Y220H | 20 | 4 | 0 | 4 | 0.000673151 | 1 |  |
| R248P | 19 | 4 | 1 | 3 | 0.000639494 | 0.75 |  |
| R156H | 19 | 4 | 2 | 2 | 0.000639494 | 0.5 |  |
| F270S | 19 | 4 | 1 | 3 | 0.000639494 | 0.75 |  |
| Y236H | 18 | 4 | 0 | 4 | 0.000605836 | 1 |  |
| I162F | 18 | 4 | 0 | 4 | 0.000605836 | 1 |  |
| G226G | 18 | 4 | 2 | 2 | 0.000605836 | 0.5 |  |
| C135R | 18 | 4 | 0 | 4 | 0.000605836 | 1 |  |
| M160I | 17 | 4 | 1 | 3 | 0.000572179 | 0.75 |  |
| G262V | 17 | 4 | 0 | 4 | 0.000572179 | 1 |  |
| E258Q | 17 | 4 | 0 | 4 | 0.000572179 | 1 |  |
| V157D | 16 | 4 | 1 | 3 | 0.000538521 | 0.75 |  |
| L130F | 16 | 4 | 0 | 4 | 0.000538521 | 1 |  |
| H168Y | 16 | 4 | 2 | 2 | 0.000538521 | 0.5 |  |
| P250P | 15 | 4 | 0 | 4 | 0.000504864 | 1 |  |
| H193D | 15 | 4 | 2 | 2 | 0.000504864 | 0.5 |  |
| C176R | 15 | 4 | 0 | 4 | 0.000504864 | 1 |  |
| A161V | 15 | 4 | 1 | 3 | 0.000504864 | 0.75 |  |
| P153P | 14 | 4 | 1 | 3 | 0.000471206 | 0.75 |  |
| P151L | 14 | 4 | 0 | 4 | 0.000471206 | 1 |  |
| M246T | 14 | 4 | 0 | 4 | 0.000471206 | 1 |  |
| H178Y | 14 | 4 | 1 | 3 | 0.000471206 | 0.75 |  |
| Y234* | 13 | 4 | 3 | 1 | 0.000437548 | 0.25 |  |
| Y205H | 13 | 4 | 0 | 4 | 0.000437548 | 1 |  |
| S241P | 13 | 4 | 0 | 4 | 0.000437548 | 1 |  |
| P142L | 13 | 4 | 2 | 2 | 0.000437548 | 0.5 |  |
| A276V | 13 | 4 | 1 | 3 | 0.000437548 | 0.75 |  |
| A276T | 13 | 4 | 0 | 4 | 0.000437548 | 1 |  |
| T284P | 12 | 4 | 0 | 4 | 0.000403891 | 1 |  |
| S269N | 12 | 4 | 2 | 2 | 0.000403891 | 0.5 |  |
| Q167H | 12 | 4 | 3 | 1 | 0.000403891 | 0.25 |  |
| M243L | 12 | 4 | 1 | 3 | 0.000403891 | 0.75 |  |
| I255N | 12 | 4 | 1 | 3 | 0.000403891 | 0.75 |  |
| S127Y | 11 | 4 | 0 | 4 | 0.000370233 | 1 |  |
| Q144R | 11 | 4 | 4 | 0 | 0.000370233 | 0 |  |
| I254T | 11 | 4 | 0 | 4 | 0.000370233 | 1 |  |
| I232F | 11 | 4 | 1 | 3 | 0.000370233 | 0.75 |  |
| G244A | 11 | 4 | 0 | 4 | 0.000370233 | 1 |  |
| E224E | 11 | 4 | 2 | 2 | 0.000370233 | 0.5 |  |
| D281Y | 11 | 4 | 0 | 4 | 0.000370233 | 1 |  |
| Y236D | 10 | 4 | 0 | 4 | 0.000336576 | 1 |  |
| V274G | 10 | 4 | 0 | 4 | 0.000336576 | 1 |  |
| N239K | 10 | 4 | 0 | 4 | 0.000336576 | 1 |  |
| L257L | 10 | 4 | 0 | 4 | 0.000336576 | 1 |  |
| L201F | 10 | 4 | 1 | 3 | 0.000336576 | 0.75 |  |
| K164N | 10 | 4 | 0 | 4 | 0.000336576 | 1 |  |
| G293G | 10 | 4 | 1 | 3 | 0.000336576 | 0.75 |  |
| G199V | 10 | 4 | 0 | 4 | 0.000336576 | 1 |  |
| G154D | 10 | 4 | 1 | 3 | 0.000336576 | 0.75 |  |
| E298K | 10 | 4 | 1 | 3 | 0.000336576 | 0.75 |  |
| E171G | 10 | 4 | 1 | 3 | 0.000336576 | 0.75 |  |
| C275W | 10 | 4 | 1 | 3 | 0.000336576 | 0.75 |  |
| S215C | 9 | 4 | 3 | 1 | 0.000302918 | 0.25 |  |
| Q136R | 9 | 4 | 1 | 3 | 0.000302918 | 0.75 |  |
| P301S | 9 | 4 | 1 | 3 | 0.000302918 | 0.75 |  |
| K292R | 9 | 4 | 2 | 2 | 0.000302918 | 0.5 |  |
| I255S | 9 | 4 | 1 | 3 | 0.000302918 | 0.75 |  |
| I162V | 9 | 4 | 1 | 3 | 0.000302918 | 0.75 |  |
| C182S | 9 | 4 | 1 | 3 | 0.000302918 | 0.75 |  |
| R283L | 8 | 4 | 2 | 2 | 0.000269261 | 0.5 |  |
| P128S | 8 | 4 | 2 | 2 | 0.000269261 | 0.5 |  |
| M133I | 8 | 4 | 0 | 4 | 0.000269261 | 1 |  |
| E224K | 8 | 4 | 2 | 2 | 0.000269261 | 0.5 |  |
| C275G | 8 | 4 | 0 | 4 | 0.000269261 | 1 |  |
| T284T | 7 | 4 | 1 | 3 | 0.000235603 | 0.75 |  |
| M133R | 7 | 4 | 0 | 4 | 0.000235603 | 1 |  |
| G262S | 7 | 4 | 2 | 2 | 0.000235603 | 0.5 |  |
| F270Y | 7 | 4 | 1 | 3 | 0.000235603 | 0.75 |  |
| E285E | 7 | 4 | 1 | 3 | 0.000235603 | 0.75 |  |
| Q165R | 6 | 4 | 2 | 2 | 0.000201945 | 0.5 |  |
| N235I | 6 | 4 | 0 | 4 | 0.000201945 | 1 |  |
| H178H | 6 | 4 | 3 | 1 | 0.000201945 | 0.25 |  |
| E204K | 6 | 4 | 2 | 2 | 0.000201945 | 0.5 |  |
| V147V | 5 | 4 | 0 | 4 | 0.000168288 | 1 |  |
| R202P | 5 | 4 | 1 | 3 | 0.000168288 | 0.75 |  |
| L130P | 5 | 4 | 2 | 2 | 0.000168288 | 0.5 |  |
| S240N | 4 | 4 | 0 | 4 | 0.00013463 | 1 |  |
| Q317K | 4 | 4 | 1 | 3 | 0.00013463 | 0.75 |  |
| E285* | 25 | 3 | 3 | 0 | 0.000841439 | 0 |  |
| L265P | 24 | 3 | 1 | 2 | 0.000807782 | 0.666666667 |  |
| S240G | 21 | 3 | 1 | 2 | 0.000706809 | 0.666666667 |  |
| E285V | 21 | 3 | 0 | 3 | 0.000706809 | 1 |  |
| V173A | 19 | 3 | 1 | 2 | 0.000639494 | 0.666666667 |  |
| R337C | 19 | 3 | 0 | 3 | 0.000639494 | 1 |  |
| T155T | 18 | 3 | 3 | 0 | 0.000605836 | 0 |  |
| G244G | 18 | 3 | 1 | 2 | 0.000605836 | 0.666666667 |  |
| D281G | 18 | 3 | 0 | 3 | 0.000605836 | 1 |  |
| V173V | 17 | 3 | 2 | 1 | 0.000572179 | 0.333333333 |  |
| T140I | 17 | 3 | 1 | 2 | 0.000572179 | 0.666666667 |  |
| P152P | 17 | 3 | 1 | 2 | 0.000572179 | 0.666666667 |  |
| K139N | 17 | 3 | 1 | 2 | 0.000572179 | 0.666666667 |  |
| E287K | 17 | 3 | 1 | 2 | 0.000572179 | 0.666666667 |  |
| A189V | 17 | 3 | 1 | 2 | 0.000572179 | 0.666666667 |  |
| V274L | 16 | 3 | 0 | 3 | 0.000538521 | 1 |  |
| I232T | 16 | 3 | 1 | 2 | 0.000538521 | 0.666666667 |  |
| I232N | 16 | 3 | 0 | 3 | 0.000538521 | 1 |  |
| C242W | 16 | 3 | 0 | 3 | 0.000538521 | 1 |  |
| C135S | 16 | 3 | 0 | 3 | 0.000538521 | 1 |  |
| R110P | 15 | 3 | 0 | 3 | 0.000504864 | 1 |  |
| G245A | 15 | 3 | 1 | 2 | 0.000504864 | 0.666666667 |  |
| G199E | 15 | 3 | 0 | 3 | 0.000504864 | 1 |  |
| T230I | 14 | 3 | 0 | 3 | 0.000471206 | 1 |  |
| S215G | 14 | 3 | 0 | 3 | 0.000471206 | 1 |  |
| P190S | 14 | 3 | 2 | 1 | 0.000471206 | 0.333333333 |  |
| V218G | 13 | 3 | 0 | 3 | 0.000437548 | 1 |  |
| M237V | 13 | 3 | 0 | 3 | 0.000437548 | 1 |  |
| I254N | 13 | 3 | 1 | 2 | 0.000437548 | 0.666666667 |  |
| G199R | 13 | 3 | 3 | 0 | 0.000437548 | 0 |  |
| V157V | 12 | 3 | 0 | 3 | 0.000403891 | 1 |  |
| T150I | 12 | 3 | 1 | 2 | 0.000403891 | 0.666666667 |  |
| S166L | 12 | 3 | 1 | 2 | 0.000403891 | 0.666666667 |  |
| S149F | 12 | 3 | 0 | 3 | 0.000403891 | 1 |  |
| R202H | 12 | 3 | 2 | 1 | 0.000403891 | 0.333333333 |  |
| Q144L | 12 | 3 | 2 | 1 | 0.000403891 | 0.333333333 |  |
| P278F | 12 | 3 | 0 | 3 | 0.000403891 | 1 |  |
| N235D | 12 | 3 | 1 | 2 | 0.000403891 | 0.666666667 |  |
| E258D | 12 | 3 | 0 | 3 | 0.000403891 | 1 |  |
| V203L | 11 | 3 | 0 | 3 | 0.000370233 | 1 |  |
| T155A | 11 | 3 | 1 | 2 | 0.000370233 | 0.666666667 |  |
| P250A | 11 | 3 | 1 | 2 | 0.000370233 | 0.666666667 |  |
| N239T | 11 | 3 | 0 | 3 | 0.000370233 | 1 |  |
| K305N | 11 | 3 | 0 | 3 | 0.000370233 | 1 |  |
| T253I | 10 | 3 | 1 | 2 | 0.000336576 | 0.666666667 |  |
| S149S | 10 | 3 | 0 | 3 | 0.000336576 | 1 |  |
| R283R | 10 | 3 | 2 | 1 | 0.000336576 | 0.333333333 |  |
| R249R | 10 | 3 | 0 | 3 | 0.000336576 | 1 |  |
| R158R | 10 | 3 | 1 | 2 | 0.000336576 | 0.666666667 |  |
| Q167R | 10 | 3 | 1 | 2 | 0.000336576 | 0.666666667 |  |
| P295L | 10 | 3 | 0 | 3 | 0.000336576 | 1 |  |
| P222P | 10 | 3 | 3 | 0 | 0.000336576 | 0 |  |
| N239Y | 10 | 3 | 0 | 3 | 0.000336576 | 1 |  |
| M243T | 10 | 3 | 0 | 3 | 0.000336576 | 1 |  |
| M160V | 10 | 3 | 1 | 2 | 0.000336576 | 0.666666667 |  |
| L299L | 10 | 3 | 2 | 1 | 0.000336576 | 0.333333333 |  |
| I162M | 10 | 3 | 2 | 1 | 0.000336576 | 0.333333333 |  |
| H168H | 10 | 3 | 1 | 2 | 0.000336576 | 0.666666667 |  |
| E11Q | 10 | 3 | 1 | 2 | 0.000336576 | 0.666666667 |  |
| C176* | 10 | 3 | 2 | 1 | 0.000336576 | 0.333333333 |  |
| A159S | 10 | 3 | 1 | 2 | 0.000336576 | 0.666666667 |  |
| S269S | 9 | 3 | 2 | 1 | 0.000302918 | 0.333333333 |  |
| R202S | 9 | 3 | 2 | 1 | 0.000302918 | 0.333333333 |  |
| Q136Q | 9 | 3 | 2 | 1 | 0.000302918 | 0.333333333 |  |
| P219L | 9 | 3 | 3 | 0 | 0.000302918 | 0 |  |
| N247S | 9 | 3 | 1 | 2 | 0.000302918 | 0.666666667 |  |
| K139K | 9 | 3 | 2 | 1 | 0.000302918 | 0.333333333 |  |
| E271V | 9 | 3 | 2 | 1 | 0.000302918 | 0.333333333 |  |
| C277* | 9 | 3 | 2 | 1 | 0.000302918 | 0.333333333 |  |
| A138A | 9 | 3 | 0 | 3 | 0.000302918 | 1 |  |
| S240I | 8 | 3 | 2 | 1 | 0.000269261 | 0.333333333 |  |
| Q192H | 8 | 3 | 1 | 2 | 0.000269261 | 0.666666667 |  |
| P219S | 8 | 3 | 2 | 1 | 0.000269261 | 0.333333333 |  |
| N247D | 8 | 3 | 0 | 3 | 0.000269261 | 1 |  |
| G187D | 8 | 3 | 1 | 2 | 0.000269261 | 0.666666667 |  |
| E349* | 8 | 3 | 0 | 3 | 0.000269261 | 1 |  |
| E285Q | 8 | 3 | 0 | 3 | 0.000269261 | 1 |  |
| C277G | 8 | 3 | 0 | 3 | 0.000269261 | 1 |  |
| V272G | 7 | 3 | 1 | 2 | 0.000235603 | 0.666666667 |  |
| V143E | 7 | 3 | 0 | 3 | 0.000235603 | 1 |  |
| T253S | 7 | 3 | 0 | 3 | 0.000235603 | 1 |  |
| T253P | 7 | 3 | 0 | 3 | 0.000235603 | 1 |  |
| R196R | 7 | 3 | 0 | 3 | 0.000235603 | 1 |  |
| Q144P | 7 | 3 | 0 | 3 | 0.000235603 | 1 |  |
| P82L | 7 | 3 | 0 | 3 | 0.000235603 | 1 |  |
| L257V | 7 | 3 | 1 | 2 | 0.000235603 | 0.666666667 |  |
| F212L | 7 | 3 | 2 | 1 | 0.000235603 | 0.333333333 |  |
| E294E | 7 | 3 | 1 | 2 | 0.000235603 | 0.666666667 |  |
| C277C | 7 | 3 | 1 | 2 | 0.000235603 | 0.666666667 |  |
| C176G | 7 | 3 | 0 | 3 | 0.000235603 | 1 |  |
| V217V | 6 | 3 | 1 | 2 | 0.000201945 | 0.666666667 |  |
| T211A | 6 | 3 | 1 | 2 | 0.000201945 | 0.666666667 |  |
| P219P | 6 | 3 | 1 | 2 | 0.000201945 | 0.666666667 |  |
| P190R | 6 | 3 | 1 | 2 | 0.000201945 | 0.666666667 |  |
| I254V | 6 | 3 | 0 | 3 | 0.000201945 | 1 |  |
| G293W | 6 | 3 | 0 | 3 | 0.000201945 | 1 |  |
| G279V | 6 | 3 | 0 | 3 | 0.000201945 | 1 |  |
| G279G | 6 | 3 | 2 | 1 | 0.000201945 | 0.333333333 |  |
| G187C | 6 | 3 | 1 | 2 | 0.000201945 | 0.666666667 |  |
| D281D | 6 | 3 | 1 | 2 | 0.000201945 | 0.666666667 |  |
| D259D | 6 | 3 | 2 | 1 | 0.000201945 | 0.333333333 |  |
| Y236Y | 5 | 3 | 0 | 3 | 0.000168288 | 1 |  |
| T304I | 5 | 3 | 0 | 3 | 0.000168288 | 1 |  |
| T231I | 5 | 3 | 1 | 2 | 0.000168288 | 0.666666667 |  |
| R283G | 5 | 3 | 0 | 3 | 0.000168288 | 1 |  |
| R202L | 5 | 3 | 1 | 2 | 0.000168288 | 0.666666667 |  |
| R175R | 5 | 3 | 0 | 3 | 0.000168288 | 1 |  |
| K164Q | 5 | 3 | 0 | 3 | 0.000168288 | 1 |  |
| G279W | 5 | 3 | 0 | 3 | 0.000168288 | 1 |  |
| E271G | 5 | 3 | 0 | 3 | 0.000168288 | 1 |  |
| E204E | 5 | 3 | 2 | 1 | 0.000168288 | 0.333333333 |  |
| C242C | 5 | 3 | 1 | 2 | 0.000168288 | 0.666666667 |  |
| A189T | 5 | 3 | 0 | 3 | 0.000168288 | 1 |  |
| Y236del | 4 | 3 | 1 | 2 | 0.00013463 | 0.666666667 |  |
| V225A | 4 | 3 | 1 | 2 | 0.00013463 | 0.666666667 |  |
| T125K | 4 | 3 | 0 | 3 | 0.00013463 | 1 |  |
| S260C | 4 | 3 | 2 | 1 | 0.00013463 | 0.333333333 |  |
| S227S | 4 | 3 | 0 | 3 | 0.00013463 | 1 |  |
| P222Q | 4 | 3 | 0 | 3 | 0.00013463 | 1 |  |
| P191T | 4 | 3 | 0 | 3 | 0.00013463 | 1 |  |
| M160K | 4 | 3 | 0 | 3 | 0.00013463 | 1 |  |
| L188V | 4 | 3 | 0 | 3 | 0.00013463 | 1 |  |
| K319E | 4 | 3 | 0 | 3 | 0.00013463 | 1 |  |
| G325E | 4 | 3 | 1 | 2 | 0.00013463 | 0.666666667 |  |
| G245N | 4 | 3 | 0 | 3 | 0.00013463 | 1 |  |
| C182Y | 4 | 3 | 1 | 2 | 0.00013463 | 0.666666667 |  |
| V73L | 3 | 3 | 0 | 3 | 0.000100973 | 1 |  |
| T230N | 3 | 3 | 0 | 3 | 0.000100973 | 1 |  |
| S185I | 3 | 3 | 0 | 3 | 0.000100973 | 1 |  |
| R337P | 3 | 3 | 0 | 3 | 0.000100973 | 1 |  |
| N131N | 3 | 3 | 1 | 2 | 0.000100973 | 0.666666667 |  |
| G226A | 3 | 3 | 0 | 3 | 0.000100973 | 1 |  |
| F54F | 3 | 3 | 1 | 2 | 0.000100973 | 0.666666667 |  |
| D228A | 3 | 3 | 1 | 2 | 0.000100973 | 0.666666667 |  |
| A84G | 3 | 3 | 0 | 3 | 0.000100973 | 1 |  |
| T125T | 25 | 2 | 2 | 0 | 0.000841439 | 0 |  |
| T155P | 23 | 2 | 0 | 2 | 0.000774124 | 1 |  |
| M133K | 23 | 2 | 0 | 2 | 0.000774124 | 1 |  |
| S215N | 20 | 2 | 2 | 0 | 0.000673151 | 0 |  |
| Y205D | 19 | 2 | 0 | 2 | 0.000639494 | 1 |  |
| W53* | 19 | 2 | 2 | 0 | 0.000639494 | 0 |  |
| Y220S | 17 | 2 | 0 | 2 | 0.000572179 | 1 |  |
| V218M | 17 | 2 | 1 | 1 | 0.000572179 | 0.5 |  |
| R156C | 17 | 2 | 1 | 1 | 0.000572179 | 0.5 |  |
| Q104* | 17 | 2 | 2 | 0 | 0.000572179 | 0 |  |
| E198K | 17 | 2 | 1 | 1 | 0.000572179 | 0.5 |  |
| L252F | 16 | 2 | 0 | 2 | 0.000538521 | 1 |  |
| C275R | 16 | 2 | 1 | 1 | 0.000538521 | 0.5 |  |
| L137L | 15 | 2 | 0 | 2 | 0.000504864 | 1 |  |
| K132M | 15 | 2 | 0 | 2 | 0.000504864 | 1 |  |
| E339* | 15 | 2 | 1 | 1 | 0.000504864 | 0.5 |  |
| M246R | 14 | 2 | 0 | 2 | 0.000471206 | 1 |  |
| L145R | 14 | 2 | 0 | 2 | 0.000471206 | 1 |  |
| T170M | 13 | 2 | 0 | 2 | 0.000437548 | 1 |  |
| L265L | 13 | 2 | 2 | 0 | 0.000437548 | 0 |  |
| L145L | 13 | 2 | 0 | 2 | 0.000437548 | 1 |  |
| C141C | 13 | 2 | 0 | 2 | 0.000437548 | 1 |  |
| V172D | 12 | 2 | 1 | 1 | 0.000403891 | 0.5 |  |
| V172A | 12 | 2 | 2 | 0 | 0.000403891 | 0 |  |
| T125M | 12 | 2 | 0 | 2 | 0.000403891 | 1 |  |
| M237K | 12 | 2 | 1 | 1 | 0.000403891 | 0.5 |  |
| K291* | 12 | 2 | 2 | 0 | 0.000403891 | 0 |  |
| F134V | 12 | 2 | 0 | 2 | 0.000403891 | 1 |  |
| E221* | 12 | 2 | 2 | 0 | 0.000403891 | 0 |  |
| V218E | 11 | 2 | 0 | 2 | 0.000370233 | 1 |  |
| V197M | 11 | 2 | 0 | 2 | 0.000370233 | 1 |  |
| T256A | 11 | 2 | 0 | 2 | 0.000370233 | 1 |  |
| T140T | 11 | 2 | 1 | 1 | 0.000370233 | 0.5 |  |
| R337L | 11 | 2 | 0 | 2 | 0.000370233 | 1 |  |
| H214Y | 11 | 2 | 1 | 1 | 0.000370233 | 0.5 |  |
| F113C | 11 | 2 | 0 | 2 | 0.000370233 | 1 |  |
| T304A | 10 | 2 | 2 | 0 | 0.000336576 | 0 |  |
| S185R | 10 | 2 | 0 | 2 | 0.000336576 | 1 |  |
| R196Q | 10 | 2 | 1 | 1 | 0.000336576 | 0.5 |  |
| P222L | 10 | 2 | 0 | 2 | 0.000336576 | 1 |  |
| P153L | 10 | 2 | 1 | 1 | 0.000336576 | 0.5 |  |
| P152T | 10 | 2 | 0 | 2 | 0.000336576 | 1 |  |
| G279R | 10 | 2 | 0 | 2 | 0.000336576 | 1 |  |
| E62* | 10 | 2 | 1 | 1 | 0.000336576 | 0.5 |  |
| E326* | 10 | 2 | 1 | 1 | 0.000336576 | 0.5 |  |
| E171K | 10 | 2 | 1 | 1 | 0.000336576 | 0.5 |  |
| Y107* | 9 | 2 | 2 | 0 | 0.000302918 | 0 |  |
| V272A | 9 | 2 | 0 | 2 | 0.000302918 | 1 |  |
| T253T | 9 | 2 | 2 | 0 | 0.000302918 | 0 |  |
| T155S | 9 | 2 | 1 | 1 | 0.000302918 | 0.5 |  |
| S241T | 9 | 2 | 0 | 2 | 0.000302918 | 1 |  |
| R174K | 9 | 2 | 0 | 2 | 0.000302918 | 1 |  |
| Q52* | 9 | 2 | 0 | 2 | 0.000302918 | 1 |  |
| N247I | 9 | 2 | 1 | 1 | 0.000302918 | 0.5 |  |
| I251V | 9 | 2 | 0 | 2 | 0.000302918 | 1 |  |
| I162S | 9 | 2 | 0 | 2 | 0.000302918 | 1 |  |
| H297Y | 9 | 2 | 0 | 2 | 0.000302918 | 1 |  |
| F270V | 9 | 2 | 0 | 2 | 0.000302918 | 1 |  |
| F134C | 9 | 2 | 0 | 2 | 0.000302918 | 1 |  |
| C135G | 9 | 2 | 1 | 1 | 0.000302918 | 0.5 |  |
| Y205* | 8 | 2 | 2 | 0 | 0.000269261 | 0 |  |
| V274I | 8 | 2 | 2 | 0 | 0.000269261 | 0 |  |
| V197E | 8 | 2 | 0 | 2 | 0.000269261 | 1 |  |
| R290R | 8 | 2 | 2 | 0 | 0.000269261 | 0 |  |
| R290L | 8 | 2 | 0 | 2 | 0.000269261 | 1 |  |
| R213P | 8 | 2 | 0 | 2 | 0.000269261 | 1 |  |
| R175P | 8 | 2 | 0 | 2 | 0.000269261 | 1 |  |
| R156G | 8 | 2 | 0 | 2 | 0.000269261 | 1 |  |
| Q144Q | 8 | 2 | 0 | 2 | 0.000269261 | 1 |  |
| Q136H | 8 | 2 | 0 | 2 | 0.000269261 | 1 |  |
| N131I | 8 | 2 | 0 | 2 | 0.000269261 | 1 |  |
| L130L | 8 | 2 | 1 | 1 | 0.000269261 | 0.5 |  |
| I232S | 8 | 2 | 1 | 1 | 0.000269261 | 0.5 |  |
| H296L | 8 | 2 | 0 | 2 | 0.000269261 | 1 |  |
| E343* | 8 | 2 | 1 | 1 | 0.000269261 | 0.5 |  |
| D208N | 8 | 2 | 0 | 2 | 0.000269261 | 1 |  |
| C229* | 8 | 2 | 2 | 0 | 0.000269261 | 0 |  |
| A84V | 8 | 2 | 1 | 1 | 0.000269261 | 0.5 |  |
| Y163Y | 7 | 2 | 1 | 1 | 0.000235603 | 0.5 |  |
| T140A | 7 | 2 | 0 | 2 | 0.000235603 | 1 |  |
| R181L | 7 | 2 | 0 | 2 | 0.000235603 | 1 |  |
| R156R | 7 | 2 | 0 | 2 | 0.000235603 | 1 |  |
| P191L | 7 | 2 | 0 | 2 | 0.000235603 | 1 |  |
| P152R | 7 | 2 | 0 | 2 | 0.000235603 | 1 |  |
| M133T | 7 | 2 | 2 | 0 | 0.000235603 | 0 |  |
| L111L | 7 | 2 | 1 | 1 | 0.000235603 | 0.5 |  |
| I255I | 7 | 2 | 0 | 2 | 0.000235603 | 1 |  |
| I232V | 7 | 2 | 1 | 1 | 0.000235603 | 0.5 |  |
| E51* | 7 | 2 | 2 | 0 | 0.000235603 | 0 |  |
| E221K | 7 | 2 | 1 | 1 | 0.000235603 | 0.5 |  |
| D184Y | 7 | 2 | 0 | 2 | 0.000235603 | 1 |  |
| C135C | 7 | 2 | 1 | 1 | 0.000235603 | 0.5 |  |
| Y220* | 6 | 2 | 2 | 0 | 0.000201945 | 0 |  |
| Y126D | 6 | 2 | 0 | 2 | 0.000201945 | 1 |  |
| Y103* | 6 | 2 | 2 | 0 | 0.000201945 | 0 |  |
| S260F | 6 | 2 | 0 | 2 | 0.000201945 | 1 |  |
| R267R | 6 | 2 | 0 | 2 | 0.000201945 | 1 |  |
| R209T | 6 | 2 | 1 | 1 | 0.000201945 | 0.5 |  |
| P301P | 6 | 2 | 0 | 2 | 0.000201945 | 1 |  |
| P295P | 6 | 2 | 1 | 1 | 0.000201945 | 0.5 |  |
| P190T | 6 | 2 | 0 | 2 | 0.000201945 | 1 |  |
| P142P | 6 | 2 | 0 | 2 | 0.000201945 | 1 |  |
| N288Y | 6 | 2 | 0 | 2 | 0.000201945 | 1 |  |
| L194I | 6 | 2 | 0 | 2 | 0.000201945 | 1 |  |
| K291N | 6 | 2 | 0 | 2 | 0.000201945 | 1 |  |
| K132T | 6 | 2 | 0 | 2 | 0.000201945 | 1 |  |
| I251T | 6 | 2 | 0 | 2 | 0.000201945 | 1 |  |
| I232I | 6 | 2 | 1 | 1 | 0.000201945 | 0.5 |  |
| H214Q | 6 | 2 | 1 | 1 | 0.000201945 | 0.5 |  |
| E287D | 6 | 2 | 0 | 2 | 0.000201945 | 1 |  |
| E271D | 6 | 2 | 1 | 1 | 0.000201945 | 0.5 |  |
| E221D | 6 | 2 | 0 | 2 | 0.000201945 | 1 |  |
| D228G | 6 | 2 | 1 | 1 | 0.000201945 | 0.5 |  |
| D228E | 6 | 2 | 1 | 1 | 0.000201945 | 0.5 |  |
| Y220D | 5 | 2 | 0 | 2 | 0.000168288 | 1 |  |
| V225I | 5 | 2 | 1 | 1 | 0.000168288 | 0.5 |  |
| V216G | 5 | 2 | 0 | 2 | 0.000168288 | 1 |  |
| V197L | 5 | 2 | 0 | 2 | 0.000168288 | 1 |  |
| V172G | 5 | 2 | 0 | 2 | 0.000168288 | 1 |  |
| V143L | 5 | 2 | 1 | 1 | 0.000168288 | 0.5 |  |
| S269C | 5 | 2 | 0 | 2 | 0.000168288 | 1 |  |
| R213G | 5 | 2 | 0 | 2 | 0.000168288 | 1 |  |
| R158S | 5 | 2 | 2 | 0 | 0.000168288 | 0 |  |
| P89S | 5 | 2 | 0 | 2 | 0.000168288 | 1 |  |
| P316P | 5 | 2 | 1 | 1 | 0.000168288 | 0.5 |  |
| M243K | 5 | 2 | 0 | 2 | 0.000168288 | 1 |  |
| M169T | 5 | 2 | 1 | 1 | 0.000168288 | 0.5 |  |
| M133V | 5 | 2 | 0 | 2 | 0.000168288 | 1 |  |
| I251I | 5 | 2 | 0 | 2 | 0.000168288 | 1 |  |
| I162N | 5 | 2 | 0 | 2 | 0.000168288 | 1 |  |
| G199* | 5 | 2 | 1 | 1 | 0.000168288 | 0.5 |  |
| G105V | 5 | 2 | 0 | 2 | 0.000168288 | 1 |  |
| F113S | 5 | 2 | 0 | 2 | 0.000168288 | 1 |  |
| D208E | 5 | 2 | 0 | 2 | 0.000168288 | 1 |  |
| D184H | 5 | 2 | 1 | 1 | 0.000168288 | 0.5 |  |
| C182R | 5 | 2 | 1 | 1 | 0.000168288 | 0.5 |  |
| Y205N | 4 | 2 | 0 | 2 | 0.00013463 | 1 |  |
| V216A | 4 | 2 | 0 | 2 | 0.00013463 | 1 |  |
| V203M | 4 | 2 | 1 | 1 | 0.00013463 | 0.5 |  |
| P77A | 4 | 2 | 0 | 2 | 0.00013463 | 1 |  |
| P223P | 4 | 2 | 0 | 2 | 0.00013463 | 1 |  |
| P142H | 4 | 2 | 0 | 2 | 0.00013463 | 1 |  |
| N210H | 4 | 2 | 2 | 0 | 0.00013463 | 0 |  |
| L330H | 4 | 2 | 2 | 0 | 0.00013463 | 0 |  |
| L264I | 4 | 2 | 0 | 2 | 0.00013463 | 1 |  |
| L257R | 4 | 2 | 0 | 2 | 0.00013463 | 1 |  |
| L188L | 4 | 2 | 0 | 2 | 0.00013463 | 1 |  |
| L145V | 4 | 2 | 0 | 2 | 0.00013463 | 1 |  |
| G266G | 4 | 2 | 2 | 0 | 0.00013463 | 0 |  |
| G112G | 4 | 2 | 0 | 2 | 0.00013463 | 1 |  |
| E62E | 4 | 2 | 1 | 1 | 0.00013463 | 0.5 |  |
| E204A | 4 | 2 | 0 | 2 | 0.00013463 | 1 |  |
| C242* | 4 | 2 | 0 | 2 | 0.00013463 | 1 |  |
| A276A | 4 | 2 | 0 | 2 | 0.00013463 | 1 |  |
| Y236S | 3 | 2 | 0 | 2 | 0.000100973 | 1 |  |
| T170S | 3 | 2 | 0 | 2 | 0.000100973 | 1 |  |
| S313S | 3 | 2 | 0 | 2 | 0.000100973 | 1 |  |
| S269G | 3 | 2 | 2 | 0 | 0.000100973 | 0 |  |
| S261S | 3 | 2 | 1 | 1 | 0.000100973 | 0.5 |  |
| R181R | 3 | 2 | 1 | 1 | 0.000100973 | 0.5 |  |
| Q38* | 3 | 2 | 2 | 0 | 0.000100973 | 0 |  |
| Q331P | 3 | 2 | 0 | 2 | 0.000100973 | 1 |  |
| Q165Q | 3 | 2 | 0 | 2 | 0.000100973 | 1 |  |
| P191P | 3 | 2 | 1 | 1 | 0.000100973 | 0.5 |  |
| P142F | 3 | 2 | 0 | 2 | 0.000100973 | 1 |  |
| N263I | 3 | 2 | 0 | 2 | 0.000100973 | 1 |  |
| N210D | 3 | 2 | 1 | 1 | 0.000100973 | 0.5 |  |
| L194V | 3 | 2 | 1 | 1 | 0.000100973 | 0.5 |  |
| K291T | 3 | 2 | 2 | 0 | 0.000100973 | 0 |  |
| K139Q | 3 | 2 | 0 | 2 | 0.000100973 | 1 |  |
| I162T | 3 | 2 | 0 | 2 | 0.000100973 | 1 |  |
| H297R | 3 | 2 | 0 | 2 | 0.000100973 | 1 |  |
| H233D | 3 | 2 | 1 | 1 | 0.000100973 | 0.5 |  |
| G117E | 3 | 2 | 0 | 2 | 0.000100973 | 1 |  |
| F212S | 3 | 2 | 0 | 2 | 0.000100973 | 1 |  |
| F212I | 3 | 2 | 0 | 2 | 0.000100973 | 1 |  |
| D228Y | 3 | 2 | 0 | 2 | 0.000100973 | 1 |  |
| C275* | 3 | 2 | 0 | 2 | 0.000100973 | 1 |  |
| A307S | 3 | 2 | 1 | 1 | 0.000100973 | 0.5 |  |
| V157A | 2 | 2 | 0 | 2 | 6.73151E-05 | 1 |  |
| T312I | 2 | 2 | 0 | 2 | 6.73151E-05 | 1 |  |
| T284K | 2 | 2 | 0 | 2 | 6.73151E-05 | 1 |  |
| S269T | 2 | 2 | 0 | 2 | 6.73151E-05 | 1 |  |
| S166S | 2 | 2 | 1 | 1 | 6.73151E-05 | 0.5 |  |
| R363R | 2 | 2 | 0 | 2 | 6.73151E-05 | 1 |  |
| R209S | 2 | 2 | 2 | 0 | 6.73151E-05 | 0 |  |
| R181S | 2 | 2 | 1 | 1 | 6.73151E-05 | 0.5 |  |
| R174M | 2 | 2 | 0 | 2 | 6.73151E-05 | 1 |  |
| P34L | 2 | 2 | 0 | 2 | 6.73151E-05 | 1 |  |
| P322R | 2 | 2 | 0 | 2 | 6.73151E-05 | 1 |  |
| P309P | 2 | 2 | 0 | 2 | 6.73151E-05 | 1 |  |
| P142A | 2 | 2 | 0 | 2 | 6.73151E-05 | 1 |  |
| N200D | 2 | 2 | 1 | 1 | 6.73151E-05 | 0.5 |  |
| K305T | 2 | 2 | 1 | 1 | 6.73151E-05 | 0.5 |  |
| H168N | 2 | 2 | 1 | 1 | 6.73151E-05 | 0.5 |  |
| G117R | 2 | 2 | 0 | 2 | 6.73151E-05 | 1 |  |
| E171V | 2 | 2 | 0 | 2 | 6.73151E-05 | 1 |  |
| D207E | 2 | 2 | 0 | 2 | 6.73151E-05 | 1 |  |
| D184D | 2 | 2 | 0 | 2 | 6.73151E-05 | 1 |  |
| A74A | 2 | 2 | 2 | 0 | 6.73151E-05 | 0 |  |
| A161P | 2 | 2 | 2 | 0 | 6.73151E-05 | 0 |  |
| P151R | 22 | 1 | 0 | 1 | 0.000740466 | 1 |  |
| I251N | 21 | 1 | 0 | 1 | 0.000706809 | 1 |  |
| C141* | 21 | 1 | 0 | 1 | 0.000706809 | 1 |  |
| Y220N | 18 | 1 | 0 | 1 | 0.000605836 | 1 |  |
| L257P | 16 | 1 | 0 | 1 | 0.000538521 | 1 |  |
| G245G | 16 | 1 | 1 | 0 | 0.000538521 | 0 |  |
| S241A | 14 | 1 | 0 | 1 | 0.000471206 | 1 |  |
| S166T | 13 | 1 | 0 | 1 | 0.000437548 | 1 |  |
| V272E | 12 | 1 | 1 | 0 | 0.000403891 | 0 |  |
| C238G | 12 | 1 | 0 | 1 | 0.000403891 | 1 |  |
| R213W | 11 | 1 | 0 | 1 | 0.000370233 | 1 |  |
| Q192R | 11 | 1 | 1 | 0 | 0.000370233 | 0 |  |
| M246K | 11 | 1 | 1 | 0 | 0.000370233 | 0 |  |
| V203E | 10 | 1 | 1 | 0 | 0.000336576 | 0 |  |
| V147I | 10 | 1 | 0 | 1 | 0.000336576 | 1 |  |
| R209K | 10 | 1 | 0 | 1 | 0.000336576 | 1 |  |
| R202C | 10 | 1 | 1 | 0 | 0.000336576 | 0 |  |
| R175S | 10 | 1 | 0 | 1 | 0.000336576 | 1 |  |
| C135* | 10 | 1 | 0 | 1 | 0.000336576 | 1 |  |
| V143G | 9 | 1 | 0 | 1 | 0.000302918 | 1 |  |
| R282L | 9 | 1 | 1 | 0 | 0.000302918 | 0 |  |
| R280* | 9 | 1 | 0 | 1 | 0.000302918 | 1 |  |
| P250T | 9 | 1 | 1 | 0 | 0.000302918 | 0 |  |
| P142S | 9 | 1 | 0 | 1 | 0.000302918 | 1 |  |
| N247T | 9 | 1 | 0 | 1 | 0.000302918 | 1 |  |
| M246L | 9 | 1 | 0 | 1 | 0.000302918 | 1 |  |
| L289L | 9 | 1 | 1 | 0 | 0.000302918 | 0 |  |
| L252P | 9 | 1 | 0 | 1 | 0.000302918 | 1 |  |
| H178D | 9 | 1 | 0 | 1 | 0.000302918 | 1 |  |
| E336* | 9 | 1 | 0 | 1 | 0.000302918 | 1 |  |
| W146R | 8 | 1 | 0 | 1 | 0.000269261 | 1 |  |
| V218A | 8 | 1 | 0 | 1 | 0.000269261 | 1 |  |
| T312S | 8 | 1 | 0 | 1 | 0.000269261 | 1 |  |
| S127P | 8 | 1 | 0 | 1 | 0.000269261 | 1 |  |
| R156L | 8 | 1 | 0 | 1 | 0.000269261 | 1 |  |
| Q136E | 8 | 1 | 0 | 1 | 0.000269261 | 1 |  |
| N288S | 8 | 1 | 0 | 1 | 0.000269261 | 1 |  |
| M169V | 8 | 1 | 1 | 0 | 0.000269261 | 0 |  |
| M160L | 8 | 1 | 1 | 0 | 0.000269261 | 0 |  |
| L201* | 8 | 1 | 1 | 0 | 0.000269261 | 0 |  |
| L111Q | 8 | 1 | 0 | 1 | 0.000269261 | 1 |  |
| G302E | 8 | 1 | 0 | 1 | 0.000269261 | 1 |  |
| G293R | 8 | 1 | 0 | 1 | 0.000269261 | 1 |  |
| E56* | 8 | 1 | 1 | 0 | 0.000269261 | 0 |  |
| D49H | 8 | 1 | 0 | 1 | 0.000269261 | 1 |  |
| D228N | 8 | 1 | 0 | 1 | 0.000269261 | 1 |  |
| C242G | 8 | 1 | 0 | 1 | 0.000269261 | 1 |  |
| Y126N | 7 | 1 | 0 | 1 | 0.000235603 | 1 |  |
| V147D | 7 | 1 | 0 | 1 | 0.000235603 | 1 |  |
| V143V | 7 | 1 | 0 | 1 | 0.000235603 | 1 |  |
| S94* | 7 | 1 | 1 | 0 | 0.000235603 | 0 |  |
| S240S | 7 | 1 | 0 | 1 | 0.000235603 | 1 |  |
| S106R | 7 | 1 | 0 | 1 | 0.000235603 | 1 |  |
| R273R | 7 | 1 | 1 | 0 | 0.000235603 | 0 |  |
| L264R | 7 | 1 | 0 | 1 | 0.000235603 | 1 |  |
| L252L | 7 | 1 | 0 | 1 | 0.000235603 | 1 |  |
| K319* | 7 | 1 | 0 | 1 | 0.000235603 | 1 |  |
| I254S | 7 | 1 | 0 | 1 | 0.000235603 | 1 |  |
| I254F | 7 | 1 | 1 | 0 | 0.000235603 | 0 |  |
| I195S | 7 | 1 | 0 | 1 | 0.000235603 | 1 |  |
| H233Y | 7 | 1 | 0 | 1 | 0.000235603 | 1 |  |
| G244R | 7 | 1 | 0 | 1 | 0.000235603 | 1 |  |
| G226S | 7 | 1 | 0 | 1 | 0.000235603 | 1 |  |
| F113V | 7 | 1 | 0 | 1 | 0.000235603 | 1 |  |
| E286V | 7 | 1 | 0 | 1 | 0.000235603 | 1 |  |
| E221E | 7 | 1 | 0 | 1 | 0.000235603 | 1 |  |
| C238W | 7 | 1 | 0 | 1 | 0.000235603 | 1 |  |
| C182* | 7 | 1 | 0 | 1 | 0.000235603 | 1 |  |
| C141F | 7 | 1 | 0 | 1 | 0.000235603 | 1 |  |
| A276G | 7 | 1 | 0 | 1 | 0.000235603 | 1 |  |
| A129V | 7 | 1 | 1 | 0 | 0.000235603 | 0 |  |
| Y234D | 6 | 1 | 0 | 1 | 0.000201945 | 1 |  |
| V217G | 6 | 1 | 0 | 1 | 0.000201945 | 1 |  |
| V217A | 6 | 1 | 1 | 0 | 0.000201945 | 0 |  |
| T256T | 6 | 1 | 0 | 1 | 0.000201945 | 1 |  |
| S240T | 6 | 1 | 0 | 1 | 0.000201945 | 1 |  |
| S166P | 6 | 1 | 0 | 1 | 0.000201945 | 1 |  |
| R342P | 6 | 1 | 0 | 1 | 0.000201945 | 1 |  |
| R174R | 6 | 1 | 1 | 0 | 0.000201945 | 0 |  |
| P309S | 6 | 1 | 0 | 1 | 0.000201945 | 1 |  |
| P223L | 6 | 1 | 0 | 1 | 0.000201945 | 1 |  |
| M237R | 6 | 1 | 0 | 1 | 0.000201945 | 1 |  |
| L194L | 6 | 1 | 0 | 1 | 0.000201945 | 1 |  |
| L194H | 6 | 1 | 0 | 1 | 0.000201945 | 1 |  |
| L130H | 6 | 1 | 0 | 1 | 0.000201945 | 1 |  |
| L111P | 6 | 1 | 0 | 1 | 0.000201945 | 1 |  |
| K320N | 6 | 1 | 0 | 1 | 0.000201945 | 1 |  |
| I254I | 6 | 1 | 1 | 0 | 0.000201945 | 0 |  |
| I251L | 6 | 1 | 0 | 1 | 0.000201945 | 1 |  |
| H233H | 6 | 1 | 1 | 0 | 0.000201945 | 0 |  |
| H179P | 6 | 1 | 0 | 1 | 0.000201945 | 1 |  |
| H178Q | 6 | 1 | 0 | 1 | 0.000201945 | 1 |  |
| G325* | 6 | 1 | 1 | 0 | 0.000201945 | 0 |  |
| G262D | 6 | 1 | 1 | 0 | 0.000201945 | 0 |  |
| E294K | 6 | 1 | 1 | 0 | 0.000201945 | 0 |  |
| E258A | 6 | 1 | 0 | 1 | 0.000201945 | 1 |  |
| E171E | 6 | 1 | 1 | 0 | 0.000201945 | 0 |  |
| D281A | 6 | 1 | 0 | 1 | 0.000201945 | 1 |  |
| D207N | 6 | 1 | 0 | 1 | 0.000201945 | 1 |  |
| D148E | 6 | 1 | 0 | 1 | 0.000201945 | 1 |  |
| C277W | 6 | 1 | 0 | 1 | 0.000201945 | 1 |  |
| C275C | 6 | 1 | 0 | 1 | 0.000201945 | 1 |  |
| A161G | 6 | 1 | 1 | 0 | 0.000201945 | 0 |  |
| Y163S | 5 | 1 | 0 | 1 | 0.000168288 | 1 |  |
| Y107Y | 5 | 1 | 1 | 0 | 0.000168288 | 0 |  |
| V31I | 5 | 1 | 1 | 0 | 0.000168288 | 0 |  |
| T231A | 5 | 1 | 1 | 0 | 0.000168288 | 0 |  |
| S183L | 5 | 1 | 1 | 0 | 0.000168288 | 0 |  |
| S149P | 5 | 1 | 0 | 1 | 0.000168288 | 1 |  |
| R280R | 5 | 1 | 1 | 0 | 0.000168288 | 0 |  |
| R202R | 5 | 1 | 0 | 1 | 0.000168288 | 1 |  |
| Q167Q | 5 | 1 | 1 | 0 | 0.000168288 | 0 |  |
| Q144H | 5 | 1 | 0 | 1 | 0.000168288 | 1 |  |
| P98S | 5 | 1 | 1 | 0 | 0.000168288 | 0 |  |
| P250F | 5 | 1 | 0 | 1 | 0.000168288 | 1 |  |
| P223H | 5 | 1 | 0 | 1 | 0.000168288 | 1 |  |
| P222S | 5 | 1 | 0 | 1 | 0.000168288 | 1 |  |
| P191S | 5 | 1 | 1 | 0 | 0.000168288 | 0 |  |
| P153T | 5 | 1 | 1 | 0 | 0.000168288 | 0 |  |
| N239N | 5 | 1 | 0 | 1 | 0.000168288 | 1 |  |
| N235Y | 5 | 1 | 0 | 1 | 0.000168288 | 1 |  |
| M133L | 5 | 1 | 0 | 1 | 0.000168288 | 1 |  |
| L308L | 5 | 1 | 1 | 0 | 0.000168288 | 0 |  |
| L265R | 5 | 1 | 0 | 1 | 0.000168288 | 1 |  |
| L265M | 5 | 1 | 0 | 1 | 0.000168288 | 1 |  |
| L201L | 5 | 1 | 0 | 1 | 0.000168288 | 1 |  |
| K292T | 5 | 1 | 0 | 1 | 0.000168288 | 1 |  |
| K164M | 5 | 1 | 0 | 1 | 0.000168288 | 1 |  |
| K120E | 5 | 1 | 0 | 1 | 0.000168288 | 1 |  |
| I255V | 5 | 1 | 1 | 0 | 0.000168288 | 0 |  |
| I195I | 5 | 1 | 1 | 0 | 0.000168288 | 0 |  |
| H179H | 5 | 1 | 1 | 0 | 0.000168288 | 0 |  |
| G226D | 5 | 1 | 0 | 1 | 0.000168288 | 1 |  |
| G187G | 5 | 1 | 0 | 1 | 0.000168288 | 1 |  |
| E285G | 5 | 1 | 0 | 1 | 0.000168288 | 1 |  |
| E258V | 5 | 1 | 0 | 1 | 0.000168288 | 1 |  |
| E221G | 5 | 1 | 1 | 0 | 0.000168288 | 0 |  |
| D281V | 5 | 1 | 0 | 1 | 0.000168288 | 1 |  |
| D259H | 5 | 1 | 0 | 1 | 0.000168288 | 1 |  |
| D259E | 5 | 1 | 1 | 0 | 0.000168288 | 0 |  |
| D207D | 5 | 1 | 0 | 1 | 0.000168288 | 1 |  |
| D186N | 5 | 1 | 0 | 1 | 0.000168288 | 1 |  |
| D148N | 5 | 1 | 0 | 1 | 0.000168288 | 1 |  |
| A189P | 5 | 1 | 1 | 0 | 0.000168288 | 0 |  |
| A189A | 5 | 1 | 0 | 1 | 0.000168288 | 1 |  |
| A119A | 5 | 1 | 1 | 0 | 0.000168288 | 0 |  |
| Y327* | 4 | 1 | 0 | 1 | 0.00013463 | 1 |  |
| Y107D | 4 | 1 | 0 | 1 | 0.00013463 | 1 |  |
| W146S | 4 | 1 | 0 | 1 | 0.00013463 | 1 |  |
| V173E | 4 | 1 | 0 | 1 | 0.00013463 | 1 |  |
| T231S | 4 | 1 | 0 | 1 | 0.00013463 | 1 |  |
| T230T | 4 | 1 | 0 | 1 | 0.00013463 | 1 |  |
| S96F | 4 | 1 | 0 | 1 | 0.00013463 | 1 |  |
| S46F | 4 | 1 | 0 | 1 | 0.00013463 | 1 |  |
| S215T | 4 | 1 | 0 | 1 | 0.00013463 | 1 |  |
| S116C | 4 | 1 | 1 | 0 | 0.00013463 | 0 |  |
| R337H | 4 | 1 | 0 | 1 | 0.00013463 | 1 |  |
| Q331H | 4 | 1 | 0 | 1 | 0.00013463 | 1 |  |
| Q165L | 4 | 1 | 1 | 0 | 0.00013463 | 0 |  |
| Q136P | 4 | 1 | 0 | 1 | 0.00013463 | 1 |  |
| P300S | 4 | 1 | 0 | 1 | 0.00013463 | 1 |  |
| P223S | 4 | 1 | 1 | 0 | 0.00013463 | 0 |  |
| P222T | 4 | 1 | 0 | 1 | 0.00013463 | 1 |  |
| P153A | 4 | 1 | 0 | 1 | 0.00013463 | 1 |  |
| N131K | 4 | 1 | 1 | 0 | 0.00013463 | 0 |  |
| L265Q | 4 | 1 | 0 | 1 | 0.00013463 | 1 |  |
| L137P | 4 | 1 | 1 | 0 | 0.00013463 | 0 |  |
| K292N | 4 | 1 | 0 | 1 | 0.00013463 | 1 |  |
| K292K | 4 | 1 | 0 | 1 | 0.00013463 | 1 |  |
| K164K | 4 | 1 | 0 | 1 | 0.00013463 | 1 |  |
| H233Q | 4 | 1 | 0 | 1 | 0.00013463 | 1 |  |
| H214D | 4 | 1 | 0 | 1 | 0.00013463 | 1 |  |
| H193N | 4 | 1 | 0 | 1 | 0.00013463 | 1 |  |
| H178R | 4 | 1 | 1 | 0 | 0.00013463 | 0 |  |
| G187V | 4 | 1 | 1 | 0 | 0.00013463 | 0 |  |
| E56K | 4 | 1 | 1 | 0 | 0.00013463 | 0 |  |
| E298V | 4 | 1 | 0 | 1 | 0.00013463 | 1 |  |
| E294G | 4 | 1 | 1 | 0 | 0.00013463 | 0 |  |
| E286D | 4 | 1 | 0 | 1 | 0.00013463 | 1 |  |
| D61N | 4 | 1 | 1 | 0 | 0.00013463 | 0 |  |
| C238C | 4 | 1 | 0 | 1 | 0.00013463 | 1 |  |
| C141S | 4 | 1 | 1 | 0 | 0.00013463 | 0 |  |
| C124R | 4 | 1 | 0 | 1 | 0.00013463 | 1 |  |
| A129D | 4 | 1 | 0 | 1 | 0.00013463 | 1 |  |
| Y126S | 3 | 1 | 0 | 1 | 0.000100973 | 1 |  |
| W146C | 3 | 1 | 0 | 1 | 0.000100973 | 1 |  |
| V122V | 3 | 1 | 1 | 0 | 0.000100973 | 0 |  |
| T150T | 3 | 1 | 0 | 1 | 0.000100973 | 1 |  |
| S269R | 3 | 1 | 0 | 1 | 0.000100973 | 1 |  |
| S261G | 3 | 1 | 0 | 1 | 0.000100973 | 1 |  |
| S260Y | 3 | 1 | 0 | 1 | 0.000100973 | 1 |  |
| S260P | 3 | 1 | 0 | 1 | 0.000100973 | 1 |  |
| S149T | 3 | 1 | 0 | 1 | 0.000100973 | 1 |  |
| S127S | 3 | 1 | 0 | 1 | 0.000100973 | 1 |  |
| R342Q | 3 | 1 | 0 | 1 | 0.000100973 | 1 |  |
| R306R | 3 | 1 | 0 | 1 | 0.000100973 | 1 |  |
| R196G | 3 | 1 | 0 | 1 | 0.000100973 | 1 |  |
| R181G | 3 | 1 | 0 | 1 | 0.000100973 | 1 |  |
| Q104H | 3 | 1 | 1 | 0 | 0.000100973 | 0 |  |
| Q100Q | 3 | 1 | 1 | 0 | 0.000100973 | 0 |  |
| P80L | 3 | 1 | 0 | 1 | 0.000100973 | 1 |  |
| P67S | 3 | 1 | 0 | 1 | 0.000100973 | 1 |  |
| P60L | 3 | 1 | 0 | 1 | 0.000100973 | 1 |  |
| P318L | 3 | 1 | 0 | 1 | 0.000100973 | 1 |  |
| P190A | 3 | 1 | 0 | 1 | 0.000100973 | 1 |  |
| P177T | 3 | 1 | 0 | 1 | 0.000100973 | 1 |  |
| P152A | 3 | 1 | 0 | 1 | 0.000100973 | 1 |  |
| N311H | 3 | 1 | 0 | 1 | 0.000100973 | 1 |  |
| N263H | 3 | 1 | 0 | 1 | 0.000100973 | 1 |  |
| N247Y | 3 | 1 | 0 | 1 | 0.000100973 | 1 |  |
| N247K | 3 | 1 | 0 | 1 | 0.000100973 | 1 |  |
| N210N | 3 | 1 | 1 | 0 | 0.000100973 | 0 |  |
| M243V | 3 | 1 | 0 | 1 | 0.000100973 | 1 |  |
| L35F | 3 | 1 | 0 | 1 | 0.000100973 | 1 |  |
| L330R | 3 | 1 | 0 | 1 | 0.000100973 | 1 |  |
| L289V | 3 | 1 | 0 | 1 | 0.000100973 | 1 |  |
| L289P | 3 | 1 | 0 | 1 | 0.000100973 | 1 |  |
| L206L | 3 | 1 | 0 | 1 | 0.000100973 | 1 |  |
| L145M | 3 | 1 | 0 | 1 | 0.000100973 | 1 |  |
| L114* | 3 | 1 | 1 | 0 | 0.000100973 | 0 |  |
| K320* | 3 | 1 | 1 | 0 | 0.000100973 | 0 |  |
| K139T | 3 | 1 | 1 | 0 | 0.000100973 | 0 |  |
| K139E | 3 | 1 | 0 | 1 | 0.000100973 | 1 |  |
| K132K | 3 | 1 | 1 | 0 | 0.000100973 | 0 |  |
| K132* | 3 | 1 | 1 | 0 | 0.000100973 | 0 |  |
| K120R | 3 | 1 | 1 | 0 | 0.000100973 | 0 |  |
| I255M | 3 | 1 | 0 | 1 | 0.000100973 | 1 |  |
| H297P | 3 | 1 | 0 | 1 | 0.000100973 | 1 |  |
| H214H | 3 | 1 | 1 | 0 | 0.000100973 | 0 |  |
| H193H | 3 | 1 | 0 | 1 | 0.000100973 | 1 |  |
| G302R | 3 | 1 | 0 | 1 | 0.000100973 | 1 |  |
| G105R | 3 | 1 | 0 | 1 | 0.000100973 | 1 |  |
| F109S | 3 | 1 | 0 | 1 | 0.000100973 | 1 |  |
| E298E | 3 | 1 | 1 | 0 | 0.000100973 | 0 |  |
| E287G | 3 | 1 | 0 | 1 | 0.000100973 | 1 |  |
| E271E | 3 | 1 | 1 | 0 | 0.000100973 | 0 |  |
| E221A | 3 | 1 | 0 | 1 | 0.000100973 | 1 |  |
| E180D | 3 | 1 | 0 | 1 | 0.000100973 | 1 |  |
| E171D | 3 | 1 | 0 | 1 | 0.000100973 | 1 |  |
| D207G | 3 | 1 | 0 | 1 | 0.000100973 | 1 |  |
| D186G | 3 | 1 | 0 | 1 | 0.000100973 | 1 |  |
| C275S | 3 | 1 | 0 | 1 | 0.000100973 | 1 |  |
| C229Y | 3 | 1 | 1 | 0 | 0.000100973 | 0 |  |
| C229R | 3 | 1 | 0 | 1 | 0.000100973 | 1 |  |
| C229C | 3 | 1 | 0 | 1 | 0.000100973 | 1 |  |
| A69G | 3 | 1 | 0 | 1 | 0.000100973 | 1 |  |
| A138D | 3 | 1 | 0 | 1 | 0.000100973 | 1 |  |
| [N247K;R248W] | 3 | 1 | 0 | 1 | 0.000100973 | 1 |  |
| Y236F | 2 | 1 | 0 | 1 | 6.73151E-05 | 1 |  |
| Y234Y | 2 | 1 | 0 | 1 | 6.73151E-05 | 1 |  |
| W146G | 2 | 1 | 0 | 1 | 6.73151E-05 | 1 |  |
| V225D | 2 | 1 | 0 | 1 | 6.73151E-05 | 1 |  |
| V147F | 2 | 1 | 0 | 1 | 6.73151E-05 | 1 |  |
| T304T | 2 | 1 | 1 | 0 | 6.73151E-05 | 0 |  |
| T230S | 2 | 1 | 0 | 1 | 6.73151E-05 | 1 |  |
| T230P | 2 | 1 | 0 | 1 | 6.73151E-05 | 1 |  |
| T125R | 2 | 1 | 0 | 1 | 6.73151E-05 | 1 |  |
| S96C | 2 | 1 | 1 | 0 | 6.73151E-05 | 0 |  |
| S90F | 2 | 1 | 1 | 0 | 6.73151E-05 | 0 |  |
| S46S | 2 | 1 | 1 | 0 | 6.73151E-05 | 0 |  |
| S46P | 2 | 1 | 0 | 1 | 6.73151E-05 | 1 |  |
| S314S | 2 | 1 | 0 | 1 | 6.73151E-05 | 1 |  |
| S303I | 2 | 1 | 0 | 1 | 6.73151E-05 | 1 |  |
| S215K | 2 | 1 | 0 | 1 | 6.73151E-05 | 1 |  |
| S185N | 2 | 1 | 0 | 1 | 6.73151E-05 | 1 |  |
| S185G | 2 | 1 | 0 | 1 | 6.73151E-05 | 1 |  |
| S127C | 2 | 1 | 1 | 0 | 6.73151E-05 | 0 |  |
| R306Q | 2 | 1 | 0 | 1 | 6.73151E-05 | 1 |  |
| R273Y | 2 | 1 | 0 | 1 | 6.73151E-05 | 1 |  |
| R273Q | 2 | 1 | 1 | 0 | 6.73151E-05 | 0 |  |
| R209I | 2 | 1 | 0 | 1 | 6.73151E-05 | 1 |  |
| Q331R | 2 | 1 | 0 | 1 | 6.73151E-05 | 1 |  |
| Q317R | 2 | 1 | 0 | 1 | 6.73151E-05 | 1 |  |
| Q165E | 2 | 1 | 0 | 1 | 6.73151E-05 | 1 |  |
| P92L | 2 | 1 | 1 | 0 | 6.73151E-05 | 0 |  |
| P67L | 2 | 1 | 1 | 0 | 6.73151E-05 | 0 |  |
| P128R | 2 | 1 | 1 | 0 | 6.73151E-05 | 0 |  |
| N288K | 2 | 1 | 0 | 1 | 6.73151E-05 | 1 |  |
| N131D | 2 | 1 | 0 | 1 | 6.73151E-05 | 1 |  |
| M243R | 2 | 1 | 1 | 0 | 6.73151E-05 | 0 |  |
| L93L | 2 | 1 | 1 | 0 | 6.73151E-05 | 0 |  |
| L344P | 2 | 1 | 0 | 1 | 6.73151E-05 | 1 |  |
| L308M | 2 | 1 | 0 | 1 | 6.73151E-05 | 1 |  |
| L206F | 2 | 1 | 0 | 1 | 6.73151E-05 | 1 |  |
| L130I | 2 | 1 | 0 | 1 | 6.73151E-05 | 1 |  |
| K321* | 2 | 1 | 0 | 1 | 6.73151E-05 | 1 |  |
| K319N | 2 | 1 | 0 | 1 | 6.73151E-05 | 1 |  |
| K291Q | 2 | 1 | 1 | 0 | 6.73151E-05 | 0 |  |
| I254M | 2 | 1 | 1 | 0 | 6.73151E-05 | 0 |  |
| I232L | 2 | 1 | 1 | 0 | 6.73151E-05 | 0 |  |
| I195V | 2 | 1 | 0 | 1 | 6.73151E-05 | 1 |  |
| H297H | 2 | 1 | 1 | 0 | 6.73151E-05 | 0 |  |
| H296H | 2 | 1 | 0 | 1 | 6.73151E-05 | 1 |  |
| H168Q | 2 | 1 | 0 | 1 | 6.73151E-05 | 1 |  |
| H168D | 2 | 1 | 0 | 1 | 6.73151E-05 | 1 |  |
| G59D | 2 | 1 | 1 | 0 | 6.73151E-05 | 0 |  |
| G293V | 2 | 1 | 0 | 1 | 6.73151E-05 | 1 |  |
| G154A | 2 | 1 | 0 | 1 | 6.73151E-05 | 1 |  |
| G108S | 2 | 1 | 0 | 1 | 6.73151E-05 | 1 |  |
| G108G | 2 | 1 | 0 | 1 | 6.73151E-05 | 1 |  |
| F341C | 2 | 1 | 0 | 1 | 6.73151E-05 | 1 |  |
| E62D | 2 | 1 | 0 | 1 | 6.73151E-05 | 1 |  |
| E343G | 2 | 1 | 1 | 0 | 6.73151E-05 | 0 |  |
| E294Q | 2 | 1 | 0 | 1 | 6.73151E-05 | 1 |  |
| E285D | 2 | 1 | 0 | 1 | 6.73151E-05 | 1 |  |
| E258E | 2 | 1 | 0 | 1 | 6.73151E-05 | 1 |  |
| E204G | 2 | 1 | 0 | 1 | 6.73151E-05 | 1 |  |
| E198Q | 2 | 1 | 0 | 1 | 6.73151E-05 | 1 |  |
| E180Q | 2 | 1 | 0 | 1 | 6.73151E-05 | 1 |  |
| E11K | 2 | 1 | 1 | 0 | 6.73151E-05 | 0 |  |
| D228H | 2 | 1 | 1 | 0 | 6.73151E-05 | 0 |  |
| D208H | 2 | 1 | 0 | 1 | 6.73151E-05 | 1 |  |
| D186V | 2 | 1 | 0 | 1 | 6.73151E-05 | 1 |  |
| D148V | 2 | 1 | 0 | 1 | 6.73151E-05 | 1 |  |
| D148D | 2 | 1 | 0 | 1 | 6.73151E-05 | 1 |  |
| C277R | 2 | 1 | 0 | 1 | 6.73151E-05 | 1 |  |
| C124S | 2 | 1 | 1 | 0 | 6.73151E-05 | 0 |  |
| A83V | 2 | 1 | 0 | 1 | 6.73151E-05 | 1 |  |
| A76V | 2 | 1 | 1 | 0 | 6.73151E-05 | 0 |  |
| A189G | 2 | 1 | 1 | 0 | 6.73151E-05 | 0 |  |
| A159G | 2 | 1 | 0 | 1 | 6.73151E-05 | 1 |  |
| [R249S;P250S] | 2 | 1 | 0 | 1 | 6.73151E-05 | 1 |  |
| [M243I;G244C] | 2 | 1 | 0 | 1 | 6.73151E-05 | 1 |  |
| [M160I;A161S] | 2 | 1 | 0 | 1 | 6.73151E-05 | 1 |  |
| Y234Q | 1 | 1 | 0 | 1 | 3.36576E-05 | 1 |  |
| Y234K | 1 | 1 | 0 | 1 | 3.36576E-05 | 1 |  |
| Y205Y | 1 | 1 | 0 | 1 | 3.36576E-05 | 1 |  |
| Y126G | 1 | 1 | 0 | 1 | 3.36576E-05 | 1 |  |
| Y107H | 1 | 1 | 0 | 1 | 3.36576E-05 | 1 |  |
| V225V | 1 | 1 | 1 | 0 | 3.36576E-05 | 0 |  |
| V225L | 1 | 1 | 0 | 1 | 3.36576E-05 | 1 |  |
| V225G | 1 | 1 | 0 | 1 | 3.36576E-05 | 1 |  |
| V216W | 1 | 1 | 0 | 1 | 3.36576E-05 | 1 |  |
| V203W | 1 | 1 | 0 | 1 | 3.36576E-05 | 1 |  |
| V197A | 1 | 1 | 0 | 1 | 3.36576E-05 | 1 |  |
| V173W | 1 | 1 | 0 | 1 | 3.36576E-05 | 1 |  |
| V10I | 1 | 1 | 0 | 1 | 3.36576E-05 | 1 |  |
| T170P | 1 | 1 | 0 | 1 | 3.36576E-05 | 1 |  |
| T155M | 1 | 1 | 1 | 0 | 3.36576E-05 | 0 |  |
| T150R | 1 | 1 | 0 | 1 | 3.36576E-05 | 1 |  |
| T125P | 1 | 1 | 0 | 1 | 3.36576E-05 | 1 |  |
| S99P | 1 | 1 | 0 | 1 | 3.36576E-05 | 1 |  |
| S96P | 1 | 1 | 0 | 1 | 3.36576E-05 | 1 |  |
| S33F | 1 | 1 | 1 | 0 | 3.36576E-05 | 0 |  |
| S315P | 1 | 1 | 0 | 1 | 3.36576E-05 | 1 |  |
| S315F | 1 | 1 | 0 | 1 | 3.36576E-05 | 1 |  |
| S314F | 1 | 1 | 0 | 1 | 3.36576E-05 | 1 |  |
| S260T | 1 | 1 | 0 | 1 | 3.36576E-05 | 1 |  |
| S240P | 1 | 1 | 0 | 1 | 3.36576E-05 | 1 |  |
| S227P | 1 | 1 | 1 | 0 | 3.36576E-05 | 0 |  |
| R335H | 1 | 1 | 1 | 0 | 3.36576E-05 | 0 |  |
| R306P | 1 | 1 | 0 | 1 | 3.36576E-05 | 1 |  |
| R283S | 1 | 1 | 1 | 0 | 3.36576E-05 | 0 |  |
| R282* | 1 | 1 | 0 | 1 | 3.36576E-05 | 1 |  |
| R280P | 1 | 1 | 0 | 1 | 3.36576E-05 | 1 |  |
| R158Y | 1 | 1 | 0 | 1 | 3.36576E-05 | 1 |  |
| R110S | 1 | 1 | 0 | 1 | 3.36576E-05 | 1 |  |
| Q331Q | 1 | 1 | 1 | 0 | 3.36576E-05 | 0 |  |
| Q192K | 1 | 1 | 0 | 1 | 3.36576E-05 | 1 |  |
| Q167K | 1 | 1 | 0 | 1 | 3.36576E-05 | 1 |  |
| Q100R | 1 | 1 | 0 | 1 | 3.36576E-05 | 1 |  |
| P98P | 1 | 1 | 0 | 1 | 3.36576E-05 | 1 |  |
| P77Q | 1 | 1 | 1 | 0 | 3.36576E-05 | 0 |  |
| P71P | 1 | 1 | 1 | 0 | 3.36576E-05 | 0 |  |
| P60P | 1 | 1 | 1 | 0 | 3.36576E-05 | 0 |  |
| P58T | 1 | 1 | 1 | 0 | 3.36576E-05 | 0 |  |
| P58L | 1 | 1 | 1 | 0 | 3.36576E-05 | 0 |  |
| P36L | 1 | 1 | 1 | 0 | 3.36576E-05 | 0 |  |
| P300R | 1 | 1 | 1 | 0 | 3.36576E-05 | 0 |  |
| P295H | 1 | 1 | 1 | 0 | 3.36576E-05 | 0 |  |
| P223R | 1 | 1 | 1 | 0 | 3.36576E-05 | 0 |  |
| P222A | 1 | 1 | 0 | 1 | 3.36576E-05 | 1 |  |
| P219H | 1 | 1 | 1 | 0 | 3.36576E-05 | 0 |  |
| P219C | 1 | 1 | 0 | 1 | 3.36576E-05 | 1 |  |
| P190H | 1 | 1 | 0 | 1 | 3.36576E-05 | 1 |  |
| P177I | 1 | 1 | 0 | 1 | 3.36576E-05 | 1 |  |
| P153H | 1 | 1 | 0 | 1 | 3.36576E-05 | 1 |  |
| P128P | 1 | 1 | 1 | 0 | 3.36576E-05 | 0 |  |
| N288D | 1 | 1 | 0 | 1 | 3.36576E-05 | 1 |  |
| N210K | 1 | 1 | 0 | 1 | 3.36576E-05 | 1 |  |
| N200N | 1 | 1 | 1 | 0 | 3.36576E-05 | 0 |  |
| M44I | 1 | 1 | 0 | 1 | 3.36576E-05 | 1 |  |
| L93M | 1 | 1 | 0 | 1 | 3.36576E-05 | 1 |  |
| L45P | 1 | 1 | 0 | 1 | 3.36576E-05 | 1 |  |
| L45M | 1 | 1 | 1 | 0 | 3.36576E-05 | 0 |  |
| L348S | 1 | 1 | 0 | 1 | 3.36576E-05 | 1 |  |
| L348F | 1 | 1 | 0 | 1 | 3.36576E-05 | 1 |  |
| L344R | 1 | 1 | 1 | 0 | 3.36576E-05 | 0 |  |
| L323R | 1 | 1 | 0 | 1 | 3.36576E-05 | 1 |  |
| L323G | 1 | 1 | 0 | 1 | 3.36576E-05 | 1 |  |
| L264Q | 1 | 1 | 0 | 1 | 3.36576E-05 | 1 |  |
| L22L | 1 | 1 | 0 | 1 | 3.36576E-05 | 1 |  |
| L206M | 1 | 1 | 0 | 1 | 3.36576E-05 | 1 |  |
| K305M | 1 | 1 | 1 | 0 | 3.36576E-05 | 0 |  |
| K305E | 1 | 1 | 0 | 1 | 3.36576E-05 | 1 |  |
| K292I | 1 | 1 | 0 | 1 | 3.36576E-05 | 1 |  |
| H365R | 1 | 1 | 0 | 1 | 3.36576E-05 | 1 |  |
| G360V | 1 | 1 | 0 | 1 | 3.36576E-05 | 1 |  |
| G356A | 1 | 1 | 0 | 1 | 3.36576E-05 | 1 |  |
| G325V | 1 | 1 | 1 | 0 | 3.36576E-05 | 0 |  |
| G293E | 1 | 1 | 0 | 1 | 3.36576E-05 | 1 |  |
| G154C | 1 | 1 | 0 | 1 | 3.36576E-05 | 1 |  |
| F338I | 1 | 1 | 0 | 1 | 3.36576E-05 | 1 |  |
| F328F | 1 | 1 | 0 | 1 | 3.36576E-05 | 1 |  |
| F113G | 1 | 1 | 0 | 1 | 3.36576E-05 | 1 |  |
| E68Q | 1 | 1 | 0 | 1 | 3.36576E-05 | 1 |  |
| E28A | 1 | 1 | 1 | 0 | 3.36576E-05 | 0 |  |
| E285A | 1 | 1 | 1 | 0 | 3.36576E-05 | 0 |  |
| E271P | 1 | 1 | 0 | 1 | 3.36576E-05 | 1 |  |
| E258L | 1 | 1 | 0 | 1 | 3.36576E-05 | 1 |  |
| E224V | 1 | 1 | 1 | 0 | 3.36576E-05 | 0 |  |
| E204Q | 1 | 1 | 0 | 1 | 3.36576E-05 | 1 |  |
| D48D | 1 | 1 | 0 | 1 | 3.36576E-05 | 1 |  |
| D42Y | 1 | 1 | 0 | 1 | 3.36576E-05 | 1 |  |
| D393N | 1 | 1 | 0 | 1 | 3.36576E-05 | 1 |  |
| D259P | 1 | 1 | 0 | 1 | 3.36576E-05 | 1 |  |
| D208I | 1 | 1 | 0 | 1 | 3.36576E-05 | 1 |  |
| D186E | 1 | 1 | 0 | 1 | 3.36576E-05 | 1 |  |
| C238H | 1 | 1 | 0 | 1 | 3.36576E-05 | 1 |  |
| C141A | 1 | 1 | 0 | 1 | 3.36576E-05 | 1 |  |
| C124W | 1 | 1 | 0 | 1 | 3.36576E-05 | 1 |  |
| A86V | 1 | 1 | 0 | 1 | 3.36576E-05 | 1 |  |
| A79G | 1 | 1 | 1 | 0 | 3.36576E-05 | 0 |  |
| A76T | 1 | 1 | 0 | 1 | 3.36576E-05 | 1 |  |
| A76G | 1 | 1 | 0 | 1 | 3.36576E-05 | 1 |  |
| A76A | 1 | 1 | 0 | 1 | 3.36576E-05 | 1 |  |
| A74V | 1 | 1 | 1 | 0 | 3.36576E-05 | 0 |  |
| A74T | 1 | 1 | 0 | 1 | 3.36576E-05 | 1 |  |
| A70A | 1 | 1 | 1 | 0 | 3.36576E-05 | 0 |  |
| A63T | 1 | 1 | 1 | 0 | 3.36576E-05 | 0 |  |
| A63P | 1 | 1 | 0 | 1 | 3.36576E-05 | 1 |  |
| A39P | 1 | 1 | 0 | 1 | 3.36576E-05 | 1 |  |
| A364T | 1 | 1 | 0 | 1 | 3.36576E-05 | 1 |  |
| A364P | 1 | 1 | 0 | 1 | 3.36576E-05 | 1 |  |
| A353T | 1 | 1 | 1 | 0 | 3.36576E-05 | 0 |  |
| A189D | 1 | 1 | 0 | 1 | 3.36576E-05 | 1 |  |
| A119D | 1 | 1 | 0 | 1 | 3.36576E-05 | 1 |  |
| [V203L/E204V] | 1 | 1 | 0 | 1 | 3.36576E-05 | 1 |  |
| [Q167Y;H168L] | 1 | 1 | 0 | 1 | 3.36576E-05 | 1 |  |
| [N29K;N30D] | 1 | 1 | 1 | 0 | 3.36576E-05 | 0 |  |
| [M160I;A161T] | 1 | 1 | 0 | 1 | 3.36576E-05 | 1 |  |
| [H168L/M169I] | 1 | 1 | 0 | 1 | 3.36576E-05 | 1 |  |
| [F270L;E271*] | 1 | 1 | 0 | 1 | 3.36576E-05 | 1 |  |

| **Table S2.** *Distribution of p53 IHC expression data among tumour sites in the IARC TP53 Database* | | | | | | |
| --- | --- | --- | --- | --- | --- | --- |
| **Tumour sites** | **Somatic TP53 mutations in IARC** | **p53 IHC available** | **Negative IHC Data** | **Positive IHC Data** | **Somatic TP53 mutation frequency** | **p53 IHC positivity** |
| LUNG | 3158 | 891 | 218 | 673 | 0.1063 | 0.755331 |
| OVARY | 2334 | 848 | 224 | 624 | 0.0786 | 0.735849 |
| BREAST | 3399 | 725 | 211 | 514 | 0.1144 | 0.708966 |
| ESOPHAGUS | 2011 | 724 | 163 | 561 | 0.0677 | 0.774862 |
| BLADDER | 1526 | 428 | 118 | 310 | 0.0514 | 0.724299 |
| BRAIN | 1783 | 359 | 58 | 301 | 0.0600 | 0.83844 |
| COLON | 1152 | 348 | 72 | 276 | 0.0388 | 0.793103 |
| COLORECTUM, NOS | 1883 | 328 | 69 | 259 | 0.0634 | 0.789634 |
| STOMACH | 1020 | 323 | 73 | 250 | 0.0343 | 0.773994 |
| SKIN | 1065 | 292 | 45 | 247 | 0.0358 | 0.84589 |
| RECTUM | 702 | 274 | 43 | 231 | 0.0236 | 0.843066 |
| LYMPH NODES | 762 | 265 | 48 | 217 | 0.0256 | 0.818868 |
| LIVER | 1217 | 239 | 56 | 183 | 0.0410 | 0.76569 |
| MOUTH^4^ | 843 | 192 | 31 | 161 | 0.0284 | 0.838542 |
| HEAD and NECK^3^ | 678 | 180 | 65 | 115 | 0.0228 | 0.638889 |
| PROSTATE | 388 | 154 | 41 | 113 | 0.0131 | 0.733766 |
| SOFT TISSUES | 435 | 130 | 47 | 83 | 0.0146 | 0.638462 |
| LARYNX | 448 | 109 | 28 | 81 | 0.0151 | 0.743119 |
| CORPUS UTERI | 223 | 108 | 23 | 85 | 0.0075 | 0.787037 |
| OROPHARYNX | 268 | 99 | 28 | 71 | 0.0090 | 0.717172 |
| PANCREAS | 483 | 84 | 25 | 59 | 0.0163 | 0.702381 |
| HEMATOP. SYSTEM | 930 | 71 | 7 | 64 | 0.0313 | 0.901408 |
| GALLBLADDER | 110 | 65 | 9 | 56 | 0.0037 | 0.861538 |
| TONGUE^5^ | 235 | 57 | 12 | 45 | 0.0079 | 0.789474 |
| ADRENAL GLAND | 95 | 55 | 20 | 35 | 0.0032 | 0.636364 |
| HYPOPHARYNX | 187 | 54 | 14 | 40 | 0.0063 | 0.740741 |
| UTERUS | 73 | 42 | 3 | 39 | 0.0025 | 0.928571 |
| NASAL CAVITY | 196 | 41 | 18 | 23 | 0.0066 | 0.560976 |
| SINUSES | 219 | 38 | 11 | 27 | 0.0074 | 0.710526 |
| BONES | 292 | 33 | 12 | 21 | 0.0098 | 0.636364 |
| CERVIX UTERI | 117 | 30 | 4 | 26 | 0.0039 | 0.866667 |
| GUM | 83 | 30 | 7 | 23 | 0.0028 | 0.766667 |
| VULVA | 125 | 27 | 6 | 21 | 0.0042 | 0.777778 |
| PAROTID GLAND | 29 | 27 | 9 | 18 | 0.0010 | 0.666667 |
| KIDNEY | 144 | 22 | 2 | 20 | 0.0048 | 0.909091 |
| RECTOSIGM. JUNCT. | 42 | 22 | 0 | 22 | 0.0014 | 1 |
| THYROID | 130 | 19 | 7 | 12 | 0.0044 | 0.631579 |
| NERVES | 80 | 13 | 4 | 9 | 0.0027 | 0.692308 |
| THYMUS | 21 | 13 | 1 | 12 | 0.0007 | 0.923077 |
| FEMALE GENITAL ORG^2^ | 28 | 12 | 0 | 12 | 0.0009 | 1 |
| SALIVARY GLAND | 22 | 12 | 2 | 10 | 0.0007 | 0.833333 |
| URETER | 26 | 11 | 2 | 9 | 0.0009 | 0.818182 |
| EYE AND ADNEXA | 29 | 10 | 2 | 8 | 0.0010 | 0.8 |
| PERITONEUM | 50 | 9 | 0 | 9 | 0.0017 | 1 |
| SMALL INTESTINE | 13 | 9 | 1 | 8 | 0.0004 | 0.888889 |
| RENAL PELVIS | 58 | 7 | 2 | 5 | 0.0020 | 0.714286 |
| UNKNOWN SITE | 26 | 7 | 1 | 6 | 0.0009 | 0.857143 |
| BILIARY TRACT | 85 | 6 | 2 | 4 | 0.0029 | 0.666667 |
| PENIS | 14 | 6 | 3 | 3 | 0.0005 | 0.5 |
| HEART/MED/PLEURA | 13 | 6 | 1 | 5 | 0.0004 | 0.833333 |
| NASOPHARYNX | 64 | 4 | 0 | 4 | 0.0022 | 1 |
| LIP | 30 | 4 | 2 | 2 | 0.0010 | 0.5 |
| TONSIL | 18 | 4 | 1 | 3 | 0.0006 | 0.75 |
| PALATE | 28 | 3 | 1 | 2 | 0.0009 | 0.666667 |
| ENDOCRINE GLANDS^1^ | 8 | 3 | 0 | 3 | 0.0003 | 1 |
| MENINGES | 2 | 2 | 0 | 2 | 0.0001 | 1 |
| TESTIS | 29 | 1 | 0 | 1 | 0.0010 | 1 |
| PYRIFORM SINUS | 12 | 1 | 0 | 1 | 0.0004 | 1 |
| SPINAL CORD | 5 | 1 | 0 | 1 | 0.0002 | 1 |
| VAGINA | 3 | 1 | 0 | 1 | 0.0001 | 1 |
| URINARY TRACT^6^ | 177 | 0 | 0 | 0 | 0.0060 | 0 |
| OTHER URINARY ORG. | 47 | 0 | 0 | 0 | 0.0016 | 0 |
| OTHER RESPIR. SYST. | 22 | 0 | 0 | 0 | 0.0007 | 0 |
| ANUS | 5 | 0 | 0 | 0 | 0.0002 | 0 |
| OTHER SITES | 4 | 0 | 0 | 0 | 0.0001 | 0 |
| OTHER DIGESTIVE ORG. | 3 | 0 | 0 | 0 | 0.0001 | 0 |
| OTHER MALE GEN. ORG. | 2 | 0 | 0 | 0 | 0.0001 | 0 |
| PLACENTA | 2 | 0 | 0 | 0 | 0.0001 | 0 |
|  |  |  |  |  |  |  |
| **Total** | **29711** | **7878** | **1852** | **6026** |  |  |
|  |  |  |  |  |  |  |
|  |  |  |  |  |  |  |
| ^1^ENDOCRINE GLANDS (ENDOCRINE GLANDS, NOS; OTHER ENDOCRINE Gl.) | | | | | | |
| ^2^FEMALE GENITAL ORG (FEMALE GENITAL ORG., NOS; OTHER FEMALE GEN. ORG.) | | | | | | |
| ^3^HEAD and NECK (HEAD&NECK, NOS; OTHER HEAD&NECK) | | | | | | |
| ^4^MOUTH (MOUTH /floor/; MOUTH /other/) | | | | | | |
| ^5^TONGUE (TONGUE /base/; TONGUE /other/) | | | | | | |
| ^6^URINARY TRACT (UP. URINARY TARCT, NOS; URINARY TRACT, NOS) | | | | | | |

|  | **Table S3.** *Comparison of mean ages of patient carrying TP53 mtations among different IHC groups* | | | | | | | | |
| --- | --- | --- | --- | --- | --- | --- | --- | --- | --- |
|  | **IHC group A** | | **IHC group B** | | **IHC group C** | | **p-values** | | |
| **Tumour sites** | **Mean age** | **SD** | **Mean age** | **SD** | **Mean age** | **SD** | **Group A vs. B** | **Group A vs. C** | **Group B vs. C** |
| BREAST | 55.06 | 13.99 | 48.83 | 13.69 | 55.97 | 14.07 | **0.020** | 0.512 | **0.008** |
| LUNG | 63.28 | 11.34 | 63.33 | 13.04 | 62.66 | 10.78 | 0.768 | 0.504 | 0.586 |
| OVARY | 55.89 | 11.11 | 59.95 | 8.87 | 55.50 | 13.33 | 0.152 | 0.929 | 0.228 |
| COLORECTUM, NOS | 66.86 | 12.88 | 66.50 | 14.42 | 65.26 | 12.77 | 0.905 | 0.198 | 0.616 |
| BRAIN | 40.66 | 17.51 | 39.46 | 16.40 | 43.04 | 18.15 | 0.883 | 0.070 | 0.320 |
| ESOPHAGUS | 59.33 | 9.85 | 61.10 | 7.59 | 58.74 | 10.62 | 0.264 | 0.708 | 0.202 |
| BLADDER | 65.90 | 12.32 | 59.52 | 14.20 | 66.44 | 12.57 | 0.094 | 0.486 | **0.019** |
| COLON | 65.07 | 12.86 | 63.68 | 9.78 | 65.29 | 12.91 | 0.208 | 0.899 | 0.184 |
| LIVER | 49.86 | 13.80 | 55.17 | 19.16 | 47.27 | 21.07 | **0.050** | 0.555 | 0.180 |
| STOMACH | 64.62 | 11.56 | 55.14 | 22.97 | 59.11 | 12.67 | 0.320 | 0.068 | 0.670 |
| HEMATOPOIETIC SYSTEM | 39.83 | 26.48 | 46.08 | 28.32 | 57.50 | 21.66 | 0.261 | **4.002E-07** | 0.264 |
| RECTUM | 63.72 | 12.26 | 68.45 | 12.78 | 66.54 | 10.15 | 0.237 | 0.270 | 0.678 |
| SKIN | 57.95 | 18.34 | 60.12 | 21.56 | 60.18 | 21.86 | 0.323 | 0.266 | 0.959 |
| LYMPH NODES | 44.10 | 23.19 | 47.80 | 16.87 | 48.86 | 21.88 | 0.772 | 0.348 | 0.796 |
| MOUTH | 60.24 | 14.29 | 61.90 | 10.68 | 58.94 | 13.39 | 0.718 | 0.696 | 0.682 |
| HEAD and NECK | 64.65 | 10.20 | 60.89 | 11.74 | 56.93 | 9.92 | 0.373 | **0.013** | 0.335 |
| Differences between mean ages of patient carrying *TP53* mtations among IHC groups were calclated by Mann-Whitney U test. Bold numbers represent significant associations (p < 0.05). | | | | | | | | | |

| **Table S4.** *Summary of nonsense mutation frequencies in different tumour sites* | | | | | | | | | | | | | | |
| --- | --- | --- | --- | --- | --- | --- | --- | --- | --- | --- | --- | --- | --- | --- |
| **Tumour sites** | **E298*** | | **Q192*** | | **R196*** | | **R213*** | | **R306*** | | **R342*** | | **W146*** | |
|  | **Frequency** | **p-value** | **Frequency** | **p-value** | **Frequency** | **p-value** | **Frequency** | **p-value** | **Frequency** | **p-value** | **Frequency** | **p-value** | **Frequency** | **p-value** |
| **BREAST** | 0.00117682 | 0.165 | 0.00647249 | **0.00822** | 0.01618123 | 0.2854 | 0.01618123 | **0.00315** | 0.0067667 | 0.3708 | 0.00500147 | 0.06785 | 0.00205943 | 0.1011 |
| **COLORECTUM, NOS** | 0 | **0.04873** | 0.00212427 | 0.3183 | 0.02018056 | 0.2346 | 0.02018056 | **0.00013** | 0.00690388 | 0.5096 | 0.00477961 | 0.2935 | 0.00371747 | 1 |
| **OVARY** | 0.0017138 | 0.611 | 0.00299914 | 0.6585 | 0.00899743 | 0.9432 | 0.00899743 | 0.3572 | 0.00771208 | 0.1847 | 0.00685518 | **0.00203** | 0.00257069 | 0.3898 |
| **ESOPHAGUS** | 0.00198906 | 0.86 | 0.00447539 | 0.7171 | 0.01193436 | 0.5157 | 0.01193436 | 0.7943 | 0.00895077 | **0.04739** | 0.00198906 | 0.4282 | 0.00596718 | 0.1553 |
| **LUNG** | 0.0079164 | **9.1E-11** | 0.00221659 | 0.181 | 0.00538315 | **0.01963** | 0.00538315 | **0.00145** | 0.00189994 | **0.00499** | 0.00094997 | **0.02744** | 0.00316656 | 0.1553 |
| **COLON** | 0.00086806 | 0.4286 | 0.00260417 | 0.6868 | 0.02430556 | **4.96E-06** | 0.02430556 | **1.8E-05** | 0.01302083 | **0.00094** | 0 | 0.08946 | 0.00260417 | 0.6208 |
| **BLADDER** | 0.00262123 | 1 | 0.01179554 | **4.1E-07** | 0.00982962 | 0.3196 | 0.00982962 | 0.7123 | 0.00065531 | **0.0133** | 0.00393185 | 0.7713 | 0.00720839 | 0.6529 |
| **SKIN** | 0.00093897 | 0.4915 | 0.00751174 | 0.07341 | 0.01314554 | **1.6E-10** | 0.01314554 | 0.6133 | 0.00093897 | 0.06272 | 0.00657277 | 0.08546 | 0.00187793 | 0.04701 |
| **STOMACH** | 0 | 0.2005 | 0.00392157 | 1 | 0.01568627 | 0.1761 | 0.01568627 | 0.1981 | 0.00490196 | 0.9425 | 0.00196078 | 0.6659 | 0.00196078 | 0.4214 |
| **RECTUM** | 0 | 0.3497 | 0 | 0.1826 | 0.01282051 | 0.05508 | 0.01282051 | 0.7938 | 0.01851852 | **7.6E-06** | 0.0014245 | 0.6128 | 0.00712251 | 0.4643 |
| **BRAIN** | 0 | 0.05727 | 0.00056085 | **0.03819** | 0.00673023 | 0.2145 | 0.00673023 | 0.08556 | 0.00504767 | 0.8936 | 0.00280426 | 0.9299 | 0.00056085 | 0.2613 |
| **MOUTH** | 0.00474496 | 0.2983 | 0.00474496 | 0.8464 | 0.0059312 | 0.3086 | 0.0059312 | 0.1932 | 0.00830368 | 0.3886 | 0.00474496 | 0.6164 | 0.00830368 | 0.03435 |
| **HEAD and NECK, NOS** | 0.01179941 | **3.1E-06** | 0.00589971 | 0.5428 | 0.00737463 | 0.5923 | 0.00737463 | 0.4461 | 0.00294985 | 0.5056 | 0.00442478 | 0.8177 | 0.00442478 | 0.0632 |
| **LIVER** | 0.00164339 | 0.7882 | 0.00410846 | 1 | 0.00164339 | **0.02843** | 0.00164339 | **0.00194** | 0.00164339 | 0.09205 | 0.00575185 | 0.174 | 0.00246508 | 1 |
| **HAEMATOPOIETIC SYSTEM** | 0 | 0.2338 | 0.00215054 | 0.5904 | 0.00645161 | 0.8212 | 0.00645161 | 0.2186 | 0.00322581 | 0.4527 | 0 | 0.1434 | 0.00322581 | 0.5779 |
| **LYMPH NODES** | 0 | 0.3138 | 0.00131234 | 0.415 | 0.01181102 | 0.674 | 0.01181102 | 0.9881 | 0.00131234 | 0.1754 | 0.00131234 | 0.5411 | 0.00393701 | 0.9697 |
| Differences between overall and tumour specific mutation frequency was compared using 2-sample test for equality of proportions with continuity correction. Bold numbers represent significant associations (p < 0.05). | | | | | | | | | | | | | | |
